# Metabolic Determinants of Electrical Failure in Ex-Vivo Canine Model of Cardiac Arrest: Evidence for the Protective Role of Inorganic Pyrophosphate

**DOI:** 10.1371/journal.pone.0057821

**Published:** 2013-03-08

**Authors:** Junko Shibayama, Tyson G. Taylor, Paul W. Venable, Nathaniel L. Rhodes, Ryan B. Gil, Mark Warren, Adam R. Wende, E. Dale Abel, James Cox, Kenneth W. Spitzer, Alexey V. Zaitsev

**Affiliations:** 1 Nora Eccles Harrison Cardiovascular Research and Training Institute, University of Utah, Salt Lake City, Utah, United States of America; 2 School of Medicine, Division of Endocrinology, Metabolism and Diabetes, Program in Molecular Medicine, University of Utah, Salt Lake City, Utah, United States of America; 3 Metabolomics Core Research Facility, University of Utah, Salt Lake City, Utah, United States of America; 4 Department of Biochemistry, University of Utah, Salt Lake City, Utah, United States of America; University of Medicine and Dentistry of New Jersey, New Jersey Medical School, United States of America

## Abstract

**Rationale:**

Deterioration of ventricular fibrillation (VF) into asystole or severe bradycardia (electrical failure) heralds a fatal outcome of cardiac arrest. The role of metabolism in the timing of electrical failure remains unknown.

**Objective:**

To determine metabolic factors of early electrical failure in an Ex-vivo canine model of cardiac arrest (VF+global ischemia).

**Methods and Results:**

Metabolomic screening was performed in left ventricular biopsies collected before and after 0.3, 2, 5, 10 and 20 min of VF and global ischemia. Electrical activity was monitored via plunge needle electrodes and pseudo-ECG. Four out of nine hearts exhibited electrical failure at 10.1±0.9 min (*early-asys*), while 5/9 hearts maintained VF for at least 19.7 min (*late-asys*). As compared to *late-asys*, *early-asys* hearts had more ADP, less phosphocreatine, and higher levels of lactate at some time points during VF/ischemia (all comparisons p<0.05). Pre-ischemic samples from *late-asys* hearts contained ∼25 times more inorganic pyrophosphate (PPi) than *early-asys* hearts. A mechanistic role of PPi in cardioprotection was then tested by monitoring mitochondrial membrane potential (ΔΨ) during 20 min of simulated-demand ischemia using potentiometric probe TMRM in rabbit adult ventricular myocytes incubated with PPi versus control group. Untreated myocytes experienced significant loss of ΔΨ while in the PPi-treated myocytes ΔΨ was relatively maintained throughout 20 min of simulated-demand ischemia as compared to control (p<0.05).

**Conclusions:**

High tissue level of PPi may prevent ΔΨm loss and electrical failure at the early phase of ischemic stress. The link between the two protective effects may involve decreased rates of mitochondrial ATP hydrolysis and lactate accumulation.

## Introduction

In the setting of out-of-hospital sudden cardiac arrest (OHSCA), asystole is “non-shockable” rhythm associated with a very poor survival to hospital discharge [1]. In recent years the incidence of asystole as the presenting rhythm in OHSCA has increased compared to the incidence of VF and reached about 40% of all cases in several studies [2]. While the rhythm preceding asystole in the setting of OHSCA usually cannot be determined, it is likely that VF is the predominant cause of hemodynamic failure in the majority of sudden cardiac deaths [3]. Thus, it is possible that a significant proportion of asystolic cardiac arrests are due to an early electrical failure occurring in the course of, and as a consequence of, untreated long-duration VF (LDVF). The mechanisms determining the speed at which asystole develops in this setting are poorly understood.

In our *ex-vivo* canine model of LDVF the occurrence of asystole is probabilistic, such that about 50% of hearts exhibit asystole within the clinically relevant time frame (∼10 min) [4]. Importantly, blockade of the ATP-sensitive K^+^ current (*I*_K-ATP_) fully prevented asystole within this time frame [4], suggesting that activation of *I*_K-ATP_ is an important factor in the ultimate electrical failure of the heart. We hypothesized that the apparently random nature of asystole in our model of LDVF may be determined by inter-subject differences in metabolic factors upstream of *I*_K-ATP_ activation.

Several studies in the past assessed metabolic alterations in ischemic [5], [6] and fibrillating hearts [7], [8]. The present work is different from those studies at least in two major aspects. First, these prior studies generally assessed relatively limited subsets of metabolites in focused approaches. In our study, we sought to achieve comprehensive profiling of energy metabolism before and during simulated cardiac arrest taking advantage of modern metabolomics techniques [9]. Second, previous studies generally treated the study subjects as a homogenous population, whereas our study exploited the native heterogeneity of the population in order to investigate correlations between metabolic profile and the rapidity of electrical depression in the course of LDVF.

Combining metabolomic techniques with analysis of cardiac excitation in the same heart allowed us to determine the key metabolites that were associated with accelerated electrical depression and asystole. Among several active metabolites and metabolic pathways during LDVF we highlighted the key role of enhanced anaerobic glycolysis and lactate accumulation in the incidence of asystole. Moreover, metabolomic screening of preischemic hearts revealed a large baseline difference in the tissue level of inorganic pyrophosphate (PPi) between hearts exhibiting early versus late asystole. Since it was previously shown in non-cardiac models that energy stored in PPi can be used for maintenance of ΔΨ in mitochondria [10], we hypothesized that PPi can prevent ΔΨ loss in cardiac myocytes during short-term anoxia. Our subsequent cellular experiments confirmed this notion. We propose a mechanism whereby the contribution of PPi in the common pool of high-energy phosphates prevents excessive stimulation of anaerobic glycolysis, lactate accumulation, and early electrical failure during LDVF. We believe that the observed cardioprotective role of PPi may be relevant under a wider range of hypoxic/ischemic conditions and therefore merits further investigation.

## Materials and Methods

### Ethics Statement

The experimental protocol conformed to the *Guide for the Care and Use of Laboratory Animals* (The National Academy Press, 8^th^ edition, 2010) and was approved by University of Utah IACUC. All surgery was performed under sodium pentobarbital anesthesia, and all efforts were made to minimize pain.

Detailed description of Methods is presented in Text S1.

### *Ex-vivo* Canine Model of LDVF

Adult mongrel dogs of either sex (25.0±1.3 kg) were premedicated with acepromazine (0.1 mL/10 kg, i.v.) and anesthetized with sodium pentobarbital (32.5 mg/kg). The hearts were quickly isolated from the chest and perfused in a Langendorff apparatus with a mixture of blood and Tyrode’s solution [11]. Immediately preceding the induction of ischemia and VF, the perfusion was switched to warm (37°C) oxygen-saturated Tyrode’s solution to ensure control over the extracellular milieu at the onset of LDVF. VF was induced by a 9V battery approximately 10 seconds before the onset of no-flow ischemia which was sustained for 20 min.

#### Electrical recordings

Local electrical activity was recorded from three plunge needle electrodes inserted into anterior left ventricular (LV) free wall. Mid-myocardial recordings from each needle were used for analysis of local VF excitation rate [11]. Asystole/severe bradycardia was defined as spontaneous VF termination observed in all recordings followed by at least 15 sec of complete electrical silence and a rhythm with rate <20 bpm for at least one minute thereafter. This definition encompasses a wide range of extremely slow and irregular rhythms observed after VF termination. In most cases however, the rhythm was close to “pure” asystole with periods of complete silence spanning several minutes (see Figure S1). For that reason and for brevity we will refer to asystole/severe bradycardia as simply asystole.

#### Experimental groups

In the control group (*control*, n = 9) the pre-LDVF Tyrode’s solution contained normal concentrations of glucose (5.5 mM) and no insulin. In the triple glucose group (*3x-glucose*, n = 6) the pre-LDVF Tyrode’s solution contained a 3-fold higher concentration of glucose (16.5 mM) plus insulin (8 IU/L). In the glucose supplement group (*glu-suppl*, n = 4) glucose levels in the blood-Tyrode mixture was maintained at ∼5.5 mM for 90 minutes prior to LDVF in order to increase the level of tissue glycogen, but the pre-LDVF Tyrode’s solution was the same as in *control*. In *defib* group (n = 6), defibrillation shocks were applied to the heart after one minute of VF in normally perfused hearts and after 15–20 minutes of LDVF. The shocks were applied at increasing strengths of 5, 10, 20, 30 and 50 J and were repeated up to 3 times at 50 J.

Depending on the time of asystole, we defined *a-posteriori* early- and late-asystole groups using cluster analysis. These were determined for the *control* group (yielding *control-early-asys* and *control-late-asys* groups), and for combined *control*, *3x-glucose* and *glu-suppl* groups (yielding *all-early-asys* and *all-late-asys* groups).

#### Tissue sampling for metabolic analysis

Tissue samples from the anterior LV subepicardium were taken at 0.3, 2, 5, 10, and 20 min after the onset of LDVF in all *control, 3x-glucose* and *glu-suppl* hearts (see Table S1 for the average actual time of tissue sampling at each time point). In addition, pre-LDVF samples were obtained in 5/9 *control* hearts, all 6 *3x-glucose* hearts, and all 4 *glu-suppl* hearts.

ATP, ADP, AMP, phosphocreatine (PCr), and inosine levels were determined by HPLC [12]. Gas chromatography/mass-spectrometry (GC/MS) was performed as described previously [13]. Myocardial glycogen content was determined as described previously [14]. Lactate, NADH and total dinucleotides (NAD^+^+NADH) were quantified using assays from BioVision (Mountain View, CA).

### ΔΨm Measurement during Anoxia in Isolated Neonatal Cardiac Myocytes

Ventricular myocytes isolated from Sprague-Dawley neonatal rats (1 day old) were incubated in the normal DMEM+horse serum at 37°C for the first 24 hours. After that, PPi (0.1 mM) was added to the media in the treatment group, whereas the control cells remained in the same normal media, and all cells were incubated for another 24 hours. Before anoxia, the cells were superfused with normal oxygenated physiological solution containing HEPES (see Text S1) at 36±0.6°C. The solution for the PPi-treated cells had the same composition, except the addition of PPi (0.1 mM). The anoxic solution contained 2-deoxyglucose instead of glucose and the level of oxygen was effectively reduced to zero by gassing with 100% nitrogen and adding oxygen scavenger sodium dithionite. The anoxic solution used for PPi-treated cells contained PPi (0.1 mM). The cultured myocytes were stained with ΔΨm-sensitive fluorescent probe TMRM (0.4 µmol/L) and were imaged using confocal microscope Zeiss LSM 5 (excitation, 543 nm; emission, above 560 nm). Four-second recordings were taken at 60-second intervals during 5 minutes of baseline conditions and 10 minutes of anoxia.

### ΔΨm Measurement during Simulated Ischemia in Adult Ventricular Myocytes

Adult ventricular myocytes were isolated from rabbit (1.8–2.5 kg) hearts by enzymatic digestion as previously described [15], [16]. After isolation, myocytes in the PPi group were kept in a normal HEPES-buffered Tyrode’s solution (see Text S1) containing 0.1 mM of PPi for 5–6.5 hours at room temperature. Control cells were kept under the same conditions but without PPi. For imaging myocytes were placed in a flow-through chamber mounted on the stage of the confocal microscope. Myocytes in control group were perfused with the normal HEPES-buffer Tyrode’s solution at flow rate of ∼4–5 ml/min. Myocytes in the PPi group were perfused with the same solution but containing 0.1 mM of PPi. Myocytes from both groups were paced at the cycle length (CL) of 280 ms for 5 min in the normal HEPES-buffer solution (tachypacing). After that myocytes were exposed to the anoxic solution, as described above, for 20 min, while continuously paced at CL = 280 ms (tachypacing+anoxia). This pacing CL was the shortest to allow all-or-none 1∶1 capture under the conditions of this experiment; thus it was the closest possible CL to emulate the conditions of ischemic VF/VT present during cardiac arrest. Three-second recordings were taken at 60-second intervals during 5 minutes of tachypacing and 20 minutes of tachypacing+anoxia. ΔΨm was measured using TMRM as described above. To quantify ΔΨ changes, electrical stimulus was temporarily stopped during each recording (for ∼3 sec), and the total TMRM fluorescence was measured in the region encompassing the entire cell except nuclei using ImageJ software. The measurements were performed during 25 min of recording (5 min of tachypacing and 20 min of tachypacing+anoxia) and normalized to the baseline value recorded at immediately before the initiation of tachypacing. The presence of cellular contractility was determined visually in cellular images taken every minute during tachypacing and tachypacing+anoxia protocols with pacing stimuli turned on. All myocytes used in this study were rod-shaped, had well-defined striations, and did not spontaneously contract.

### Statistical Analysis

*K-means* cluster analysis was used to determine the optimal separation between the early asystole and the late asystole *a posteriori* groups. Timing of asystole was compared between *control*, *3x-glucose,* and *glu-suppl* groups using log-rank test applied to Kaplan-Meier survival curves. For all comparisons involving multiple time points we used 2-way repeated measures ANOVA with post-hoc Bonferroni test. Otherwise, two-tailed unpaired t-test was used. A value of p<0.05 was considered statistically significant. Data are given as mean ± SEM.

## Results

### Evolution of Electrical Activity and Energy Metabolism during LDVF under Baseline Conditions

#### Electrical activity and timing of asystole

**Figure 1:**
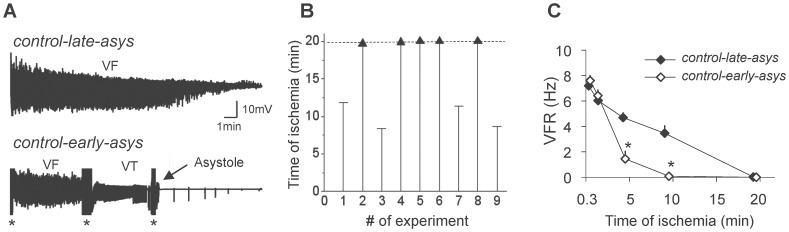
Bimodal distribution of electrophysiological outcomes in *control* group. **A.** Representative examples of LV unipolar electrograms from two hearts exhibiting late asystole (*Top*) and early asystole (*Bottom*). In early asystole a typical sequence included VF followed by ventricular tachycardia (VT) followed by asystole and a few agonal indeoventricular beats. Asterisks denote artifacts sometimes caused by tissue sampling. **B.** Distribution of asystole times in the *control* group. Asystole was observed in 4/9 hearts whereas LDVF was maintained throughout the tested interval (20 min) in 5/9 hearts. According to these outcomes, the *control* group was subdivided into *control-early-asys* and *control-late-asys groups*, respectively. **C.** The average time course of VFR measured in LV mid-myocardium during LDVF in *control-early-asys* and *control-late-asys* hearts (*:p<0.05).

In the *control* group (n = 9), 4 hearts underwent asystole relatively early in the course of LDVF (between 8.7 and 11.8 min, *control-early-asys*) whereas the remaining 5 hearts maintained VF beyond 19.7 min of ischemia (*control-late-asys*). Examples of LV unipolar electrograms from asystolic and non-asystolic hearts are shown in Figure 1A; distribution of asystole times is shown in Figure 1B. Figure 1C shows the time course of VF rate (VFR) recorded from the mid-myocardial layer in *control-early-asys* and *control-late-asys* hearts. The reduction of VFR during the first 2 min was moderate and almost identical in the two groups. However, after that VFR rapidly decreased in *control-early-asys* reaching 1.4±0.9 Hz at 5 min and approaching zero at 10 min (0.24±0.19 Hz). In contrast, in *control-late-asys* VFR was 4.6±0.6 Hz at 5 min and 3.3±1.0 Hz at 10 min (p<0.01 vs. *control-early-asys*), and approached zero only at 20 min. Endocardial electrograms exhibited similar degree of divergence in VFR between the two groups (not shown). *Control-early-asys* and *control-late-asys* hearts were not significantly different in terms of animals’ weight, sex, and other experimental parameters (see Table S2).

In *defib* group, VF was successfully terminated during normal perfusion in all hearts at the shock strength between 10 and 20 J. During LDVF, in 3/6 hearts application of shocks occurred during maintained VF (*defib-late-asys*) and was successful in all 3 hearts in the range of 20–50 J. In the remaining 3/6 hearts in which shocks occurred after the onset of asystole (*defib-early-asys)*, they elicited no response at the shock strengths up to 50 J. In 5 additional experiments LV epicardial pacing elicited no response in all hearts and LV endocardial pacing elicited response in only 1 out of 5 hearts after the onset of asystole in the course of LDVF (Taylor, Venable, Zaitsev, unpublished observations). These data indicate that asystole in our model was a non-shockable and non-paceable rhythm confirming the state of severe electrical failure and recapitulating the main feature of the clinical phenomenon.

#### High-energy phosphate (HEP) and adenine nucleotide compounds

**Figure 2:**
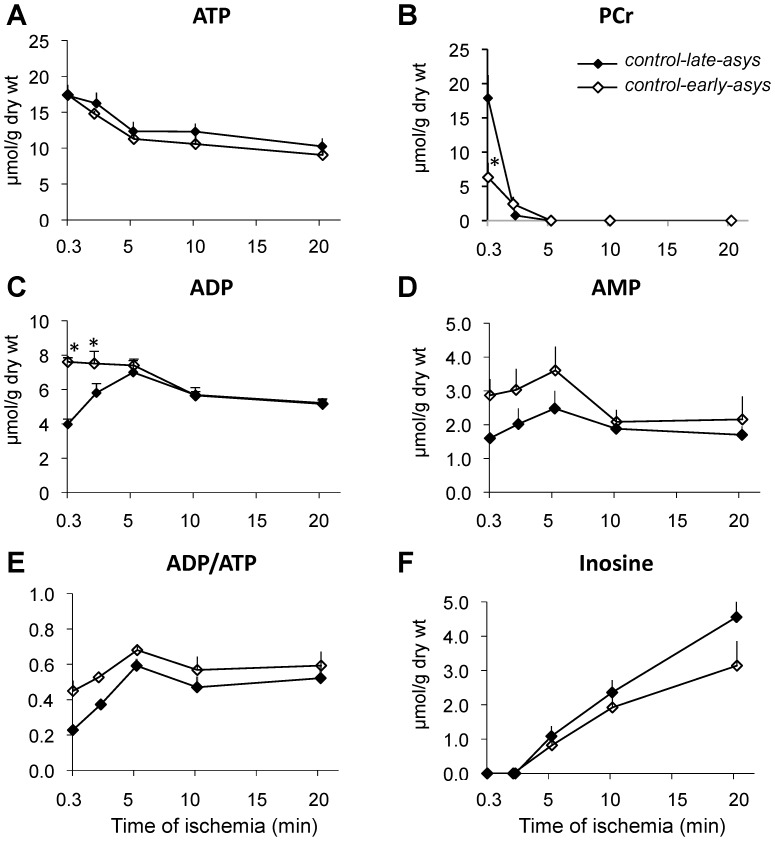
Levels of HEP and related adenine nucleotides during LDVF in *control-early-asys* and *control-late-asys* hearts. See text for detail (*:p<0.05).

Levels of HEP and adenine nucleotides as a function of ischemia time are shown in Figure 2. The levels of ATP at the earliest measured time point of LDVF (0.3 min) were not different between *control-early-asys* and *control-late-asys* hearts (17.4±0.4 µmol/g vs. 17.3±1.5 µmol/g, Figure 2A). Throughout 20 min of ischemia, ATP levels gradually declined in both *control-early-asys* and *control-late-asys* at almost identical rates, which was relatively fast between 0.3 and 5 min, but slowed down between 5 and 20 min of ischemia. There were no statistical differences in ATP levels between *control-early-asys* and *control-late-asys* at all measured time points.

In contrast to ATP, the other two HEP – PCr and ADP - showed large differences between *control-early-asys* and *control-late-asys*. At 0.3 min of LDVF, *control-early-asys* hearts had significantly lower levels of PCr than *control-late-asys* hearts (6.3±2.1 µmol/g vs. 17.9±3.4 µmol/g, respectively, p<0.0001), followed by almost complete depletion at 2 min and disappearance at 5 min in both *control-early-asys* and *control-late-asys*. *Control-early-asys* hearts also exhibited significantly higher levels of ADP than *control-late-asys* hearts at 0.3 and 2 min of LDVF. In *control-late-asys* hearts, ADP levels reached the maximum level of ADP in *control-*e*arly-asys* hearts only at 5 min of LDVF, after which point both *control-early-asys* and *control-late-asys* showed a small reduction in ADP between 5 and 10 min, followed by an almost constant level between 10 and 20 min (Figure 2C). AMP levels and ADP/ATP ratios trended higher in *control-early-asys* than in *control-late-asys* (see Figures 2D and 2E), but in neither case did the difference reach statistical significance.

Concomitant with reductions in the total adenosine nucleotide pool (ΣAd: Σ(ATP+ADP+AMP), see Figure S2), we detected an increasing level of inosine (Figure 2F), suggesting activation of adenine nucleotide degradation pathways [17]. Nevertheless, neither ΣAd nor inosine were statistically different between *control-early-asys* and *control-late-asys* at any time points of LDVF. In summary, relative to *control-late-asys* hearts, *control-early-asys* hearts exhibited an energy imbalance very early in the course of LDVF, manifested by reduced PCr level and increased ADP level, despite similar levels of ATP.

#### Glycolytic substrates, intermediates, and end-products

**Figure 3:**
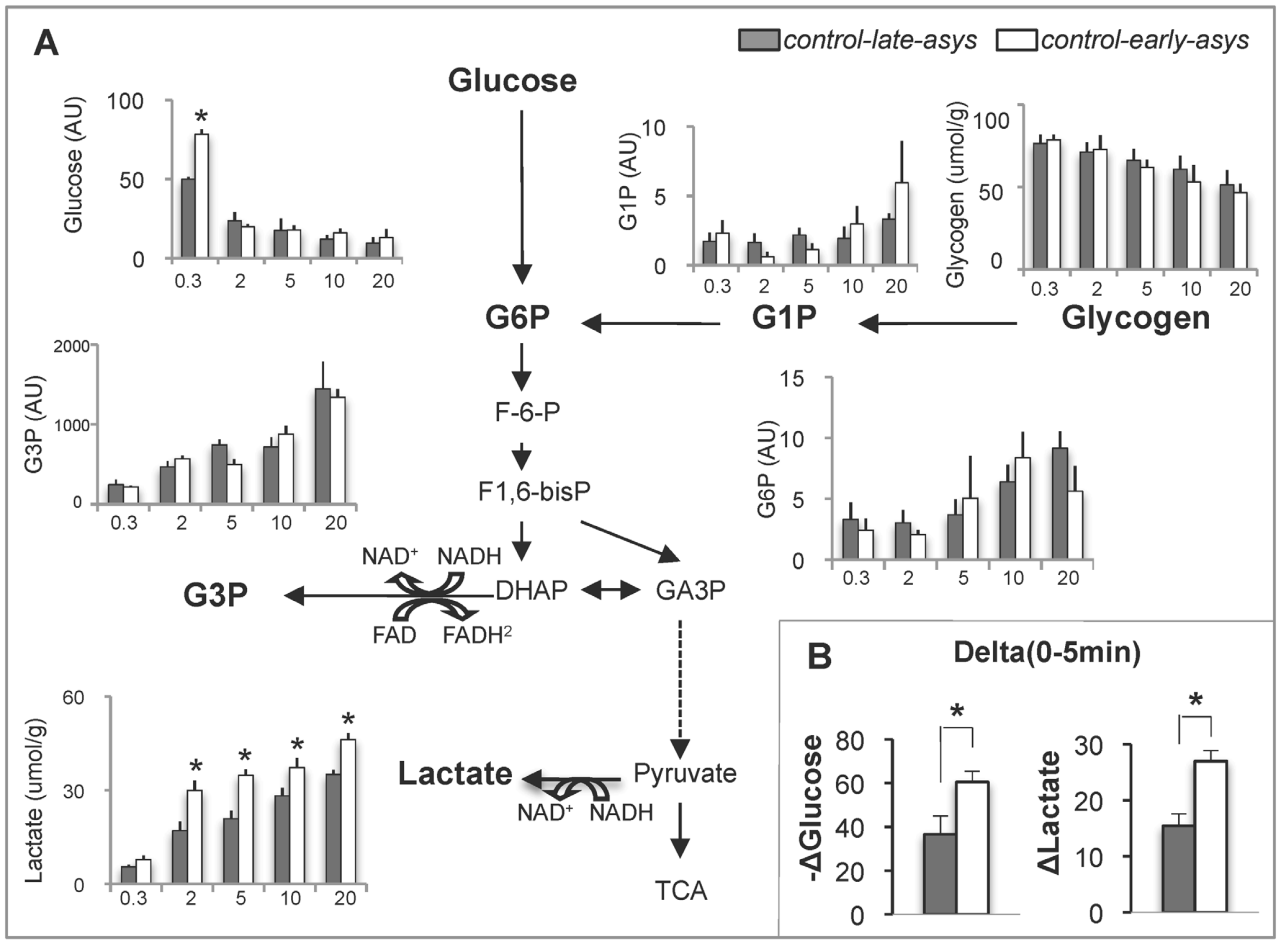
Glycolytic substrates and intermediates during LDVF in *control-early-asys* and *control-late-asys* hearts. **A.** Changes in glycolytic metabolites over time. For clarity, the data are presented as bar graphs, and x-axis indicates the approximate time of ischemia (min). G1P, glucose-1-phosphate; G6P, glucose-6-phosphate, G3P, glycerol 3-phosphate. **B.** ΔLactate and -Δglucose, the absolute difference in lactate and glucose between 0.3 and 5 min of LDVF, respectively. (*:p<0.05).

To test the role of anaerobic glycolysis in the timing of asystole, we compared the levels of glycolytic substrates and intermediates between *control-late-asys* and *control-early-asys* as summarized in Figure 3. The level of glycogen gradually decreased over the course of LDVF, but was not significantly different between *control-early-asys* and *control-late-asys* at all time points analyzed. Paradoxically, *control-early-asys* hearts exhibited a significantly higher level of glucose than *control-late-asys* hearts at 0.3 min of LDVF (p<0.0001), but due to faster consumption of glucose by *control-early-asys* hearts glucose content was not different between *control-early-asys* and *control-late-asys* after 2 min of LDVF.

Importantly, concomitant with a large reduction of glucose between 0.3 and 5 min of LDVF, the amount of lactate accumulated in the same time interval was much larger in *control-early-asys* hearts than *control-late-asys* hearts (Figure 3B). In fact, the lactate level was significantly higher in *control-early-asys* than in *control-late-asys* at all time points between 2 and 20 min of LDVF (see lactate plot in Figure 3A). Other glycolytic intermediates were not significantly different between *control-early-asys* and *control-late-asys* hearts. Likewise, the levels of NAD^+^/NADH were not significantly different between *control-early-asys* and *control-late-asys* throughout 20 min of LDVF (see Figure S3A). Thus, the single most prominent difference between *control-early-asys* and *control-late-asys* hearts was the much higher rate of lactate production in the former, apparently driven by a higher rate of glycolytic metabolism.

#### Tricarboxylic acid (TCA) cycle intermediates

**Figure 4:**
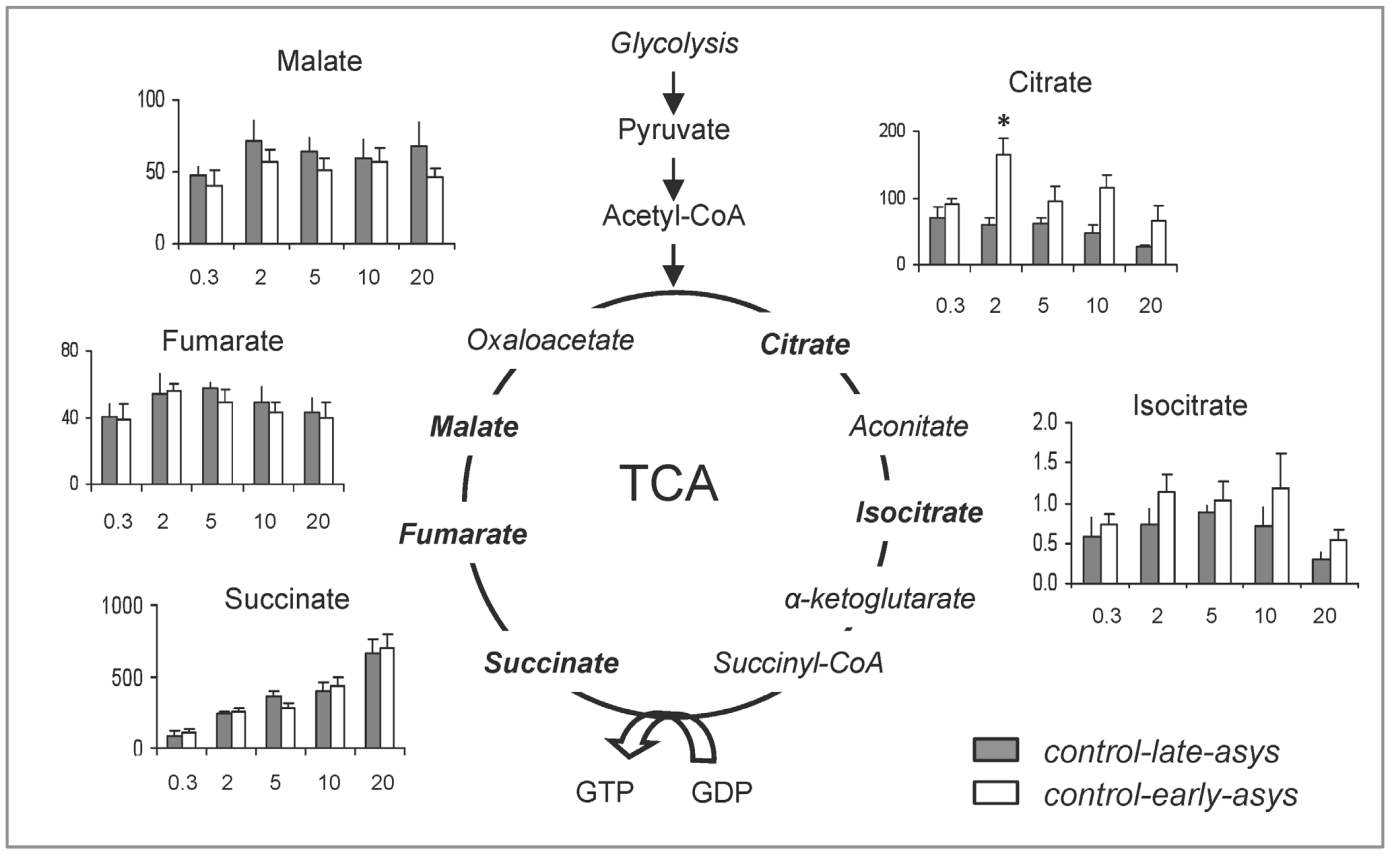
Changes in the TCA cycle intermediates during LDVF in *control-early-asys* and *control-late-asys* hearts. Values are indicated as arbitrary units (y-axis). For clarity, the data are presented as bar graphs, and x-axis indicates the approximate time of ischemia (min). (*:p<0.05).

The dynamics of TCA cycle intermediates during 20 min of LDVF are summarized in Figure 4 (Expanded version of Figure 4 including some metabolic pathways interconnected with TCA cycle is presented in Figure S4). The only significant difference between *control-early-asys* and *control-late-asys* hearts was observed in the level of citrate (at 2 min of LDVF). The most plausible source of this difference is acetyl-CoA from pyruvate, which would be consistent with the evidence of enhanced anaerobic glycolysis shown above.

### Effects of Glycolytic Substrates Manipulations

Since in the *control* group early asystole was associated with accelerated glucose utilization and accumulation of lactate (see Figure 3), we hypothesized that deliberate enhancement of glucose metabolism by increasing either free glucose or glycogen content might promote asystole in our model. The effects of stimulated glucose uptake (*3x-glucose* group) and increased glycogen content (*glu-suppl* group) prior to LDVF are shown in Figures S5, S6, S7. In *3x-glucose* group the higher level of glucose prior to LDVF led to an increased lactate accumulation and accelerated asystole as compared to *control* group (see Figure S5). In contrast, higher pre-ischemic content of glycogen in *glu-suppl* group did not cause a significant difference in the lactate accumulation and incidence of asystole as compared to *control* group (see Figure S6). Importantly, however, both these additional groups exhibited bimodal distribution of asystole times (early, before 8 min of LDVF and late, after 16 min of LDVF). Thus it appears that substrate manipulations modulated, but did not override, the innate dichotomy of outcomes in this model.

### Bimodal Distribution of Asystole and Lactate Accumulation

**Figure 5:**
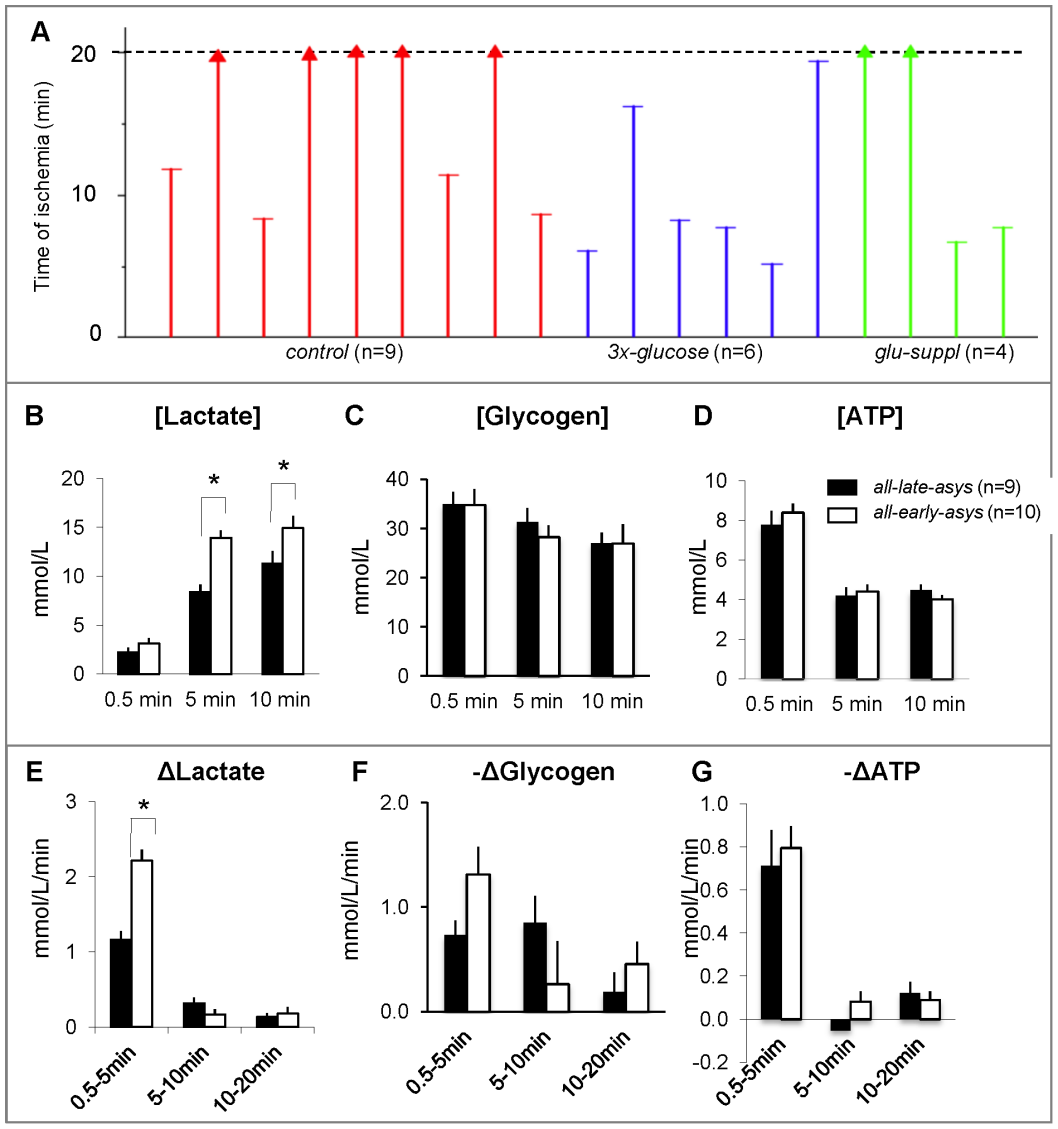
Bimodal distribution of asystole and lactate accumulation. A. Asystole time in all *control*, *3x-glucose* and *glu-suppl* groups (total n = 19). Note bimodal distribution of asystole times (early: <11.8 min, late:>16.2 min) despite manipulations on glycolytic substrates. In panels B-G, *all-early-asys* hearts (white) and *all-late-asys* hearts (black) are pooled together for metabolic comparisons. **B–D.** The absolute levels (in mM) of tissue lactate, glycogen and ATP, respectively, at different time points of LDVF. **E–G.** the rates of change (in mM/min) of lactate, glycogen and ATP, respectively, at different time intervals during LDVF. The high level of lactate at 5 and 10 min was a metabolic signature of early asystole. (*:p<0.05).

Assuming that the bimodal distribution of the asystole time was present despite substrate manipulations, we pooled together data from *control* (n = 9); *3x-glucose* (n = 6); and *glu-suppl* (n = 4) groups. Figure 5A shows timing of asystole in all 19 hearts. K-means cluster analysis revealed two clusters in the pooled data (asystole time <11.8 min and asystole time >16.2 min), which were designated as *all-early-asys* and *all-late-asys* groups, respectively. The distribution between the two groups was 53% and 47%, respectively. *All-early-asys* hearts had significantly higher levels of lactate at 5 and 10 min of LDVF than *all-late-asys* hearts (Figure 5B). In contrast, the two metabolites often considered the most important factors of ischemic metabolism (ATP and glycogen) were not predictive of early asystole in our model of LDVF (Figure 5C–D).

### Metabolomic Screening in pre-LDVF Hearts

**Figure 6:**
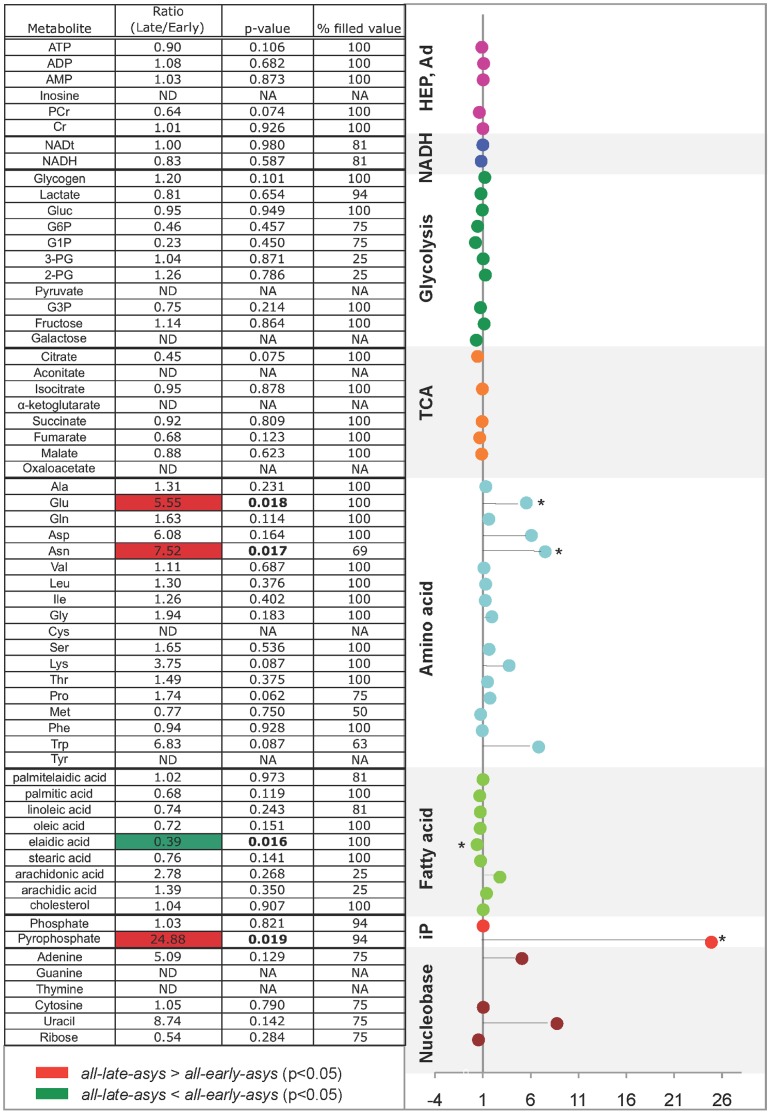
Heat map (*Left*) and fold expression (*Right*) of metabolomic screening of pre-LDVF hearts. For % filled value, the number of samples, in which each metabolite was detected, was divided by the total number of baseline samples (n = 15). For fold expression, x-axis indicates the ratio of the levels of metabolites in late-asys over those from early-asys hearts. (*:p<0.05).

The robust presence of bimodal distribution of asystole times in our model of LDVF irrespective of substrate manipulation could indicate a difference in baseline metabolism between *all-early-asys* and *all-late-asys* hearts. To test this hypothesis, we performed metabolomic screening in pre-LDVF hearts. We analyzed total of 67 metabolites, and calculated the ratio of each metabolite in *all-late-asys* hearts over that in *all-early-asys* hearts. The results are shown in Figure 6. There were no significant differences in the levels of HEP (ATP, ADP, PCr), AMP, NADH, NADtotal, glycolytic substrates and intermediates, and inorganic phosphate. Among the TCA intermediates (*orange*), the level of citrate trended lower in *all-late-asys* than in *all-early-asys* hearts (p = 0.075). Moreover, two amino acids (glutamate and asparagine) were significantly higher in *all-late-asys* than in *all-early-asys* hearts (p = 0.018 and 0.017, respectively). However, the most remarkable difference was found in the pre-ischemic level of pyrophosphate (PPi), which was 24.9 times higher in *all-late-asys* than in *all-early-asys* hearts (p = 0.019).

### Role of PPi in the Maintenance of Mitochondrial Membrane Potential (ΔΨm) during Anoxic Stress

**Figure 7:**
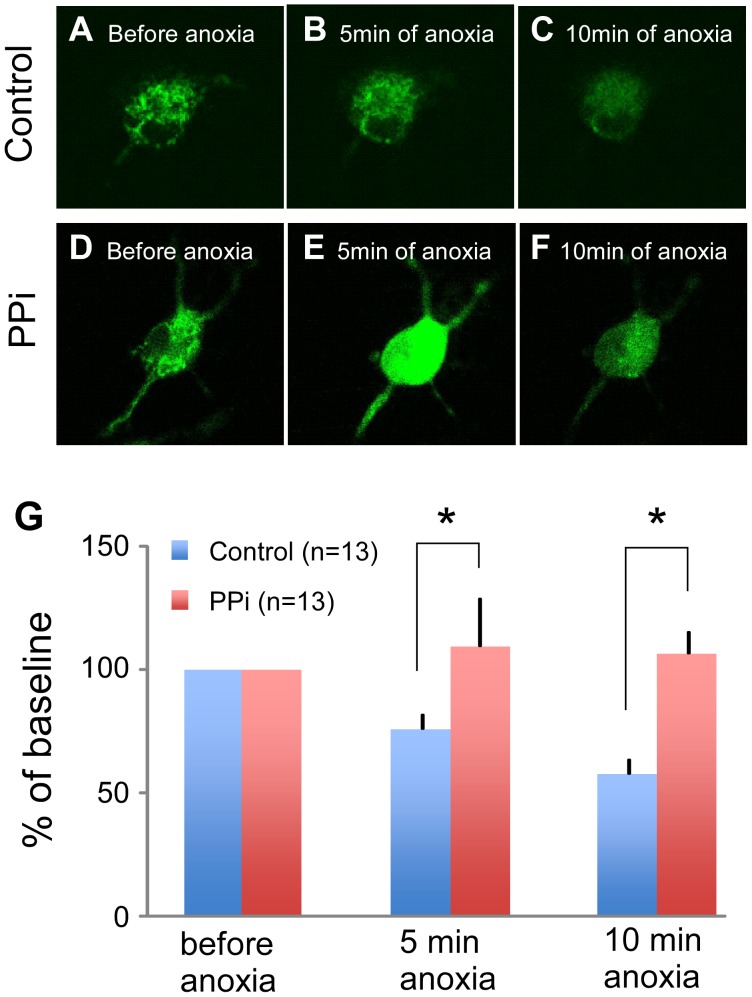
Effect of PPi on ΔΨm during near-anoxia in cultured rat neonatal cells. **A–C**. Control. ΔΨm-dependent signal gradually decreased throughout 10 min of anoxia. **D–F.** PPi-treated cell. During the first 5 min of anoxia the PPi-treated cell showed the transient increase of the TMRM fluorescence, indicating the hyperpolarization of ΔΨm, which was followed by attenuated ΔΨm depolarization between 5 and 10 min. **G.** Quantitative analysis of the TMRM signals.

It has been suggested that PPi may contribute to the maintenance of ΔΨm and ATP synthesis under various metabolic conditions [10], [18]. Thus, we hypothesized that the high content of PPi in myocytes might prevent loss of ΔΨm during anoxic stress. To test this hypothesis, we monitored ΔΨm-sensitive fluorescence of TMRM during 10 min of near-anoxia in rat neonatal cardiomyocytes incubated overnight with 0.1 mM of PPi versus control group. As shown in Figure 7A–C, myocytes in control group experienced significant loss of ΔΨm-dependent fluorescence during 10 min of anoxia (76.0±5.4% of baseline at 5 min and 57.6±6.0% of baseline at 10 min of anoxia, also see Panel G). In contrast, in the PPi-treated myocytes ΔΨm was either unchanged or slightly hyperpolarized during anoxia (109.5±19.4% of baseline at 5 min and 106.6±8.6% at 10 min of anoxia, Figure 7D–F and G).

**Figure 8:**
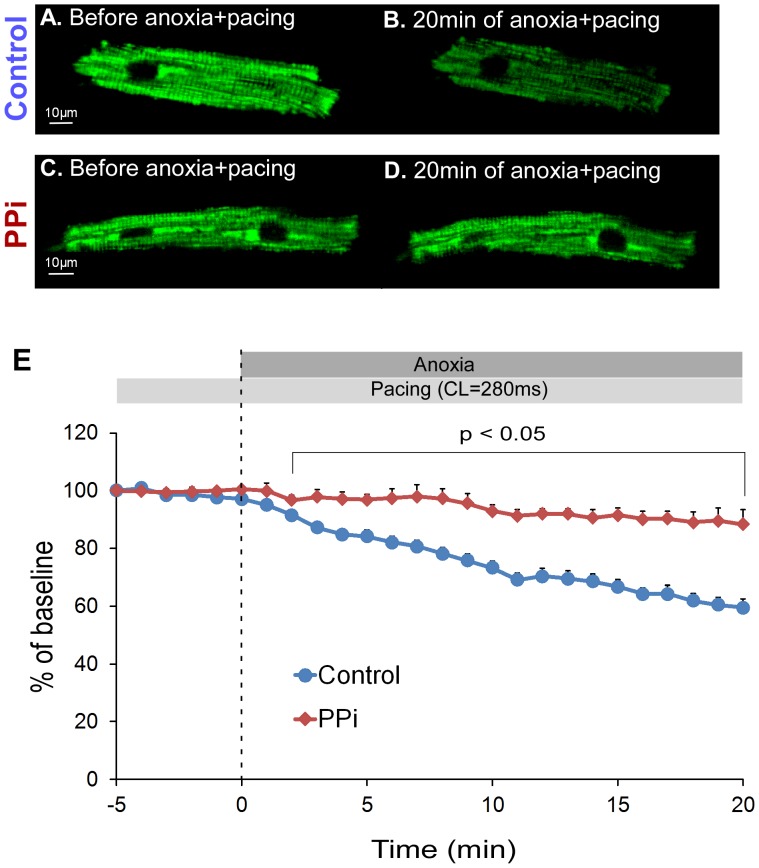
Effect of PPi on ΔΨm during anoxia+tachypacing in rabbit adult ventricular myocytes. The myocytes were paced at CL = 280 ms for 5 min in a normal HEPES-buffered perfusion solution. After that myocytes were exposed to 20 min of anoxia with continuous pacing at CL = 280 ms (anoxia+tachypacing). **A** and **B**. The image of TMRM fluorescence immediately before and after 20 min of anoxia+tachypacing in a representative control myocyte, respectively. Note that after 20 min of anoxia+tachypacing the brightness of ΔΨm-dependent TMRM signal markedly decreased. **C** and **D.** The image of TMRM fluorescence immediately before and after 20 min of anoxia+tachypacing in a representative PPi-treated myocyte, respectively. Note that the brightness of ΔΨm-dependent TMRM signal was largely preserved during 20 min of anoxia+tachypacing in this myocyte. **E.** Statistical analysis of the TMRM fluorescence during 5 minutes of tachypacing followed by 20 minutes of tachypacing+anoxia in control (n = 9) and PPi-treated (n = 7) myocytes. The measurements were normalized to the baseline value recorded at immediately before the initiation of tachypacing (at the time point “−5 min”). ΔΨm-dependent TMRM signal was significantly different between control and PPi groups at all time points between 2 and 20 of tachypacing+anoxia (p<0.05).

In order to assess the role of PPi under conditions more closely resembling VF-induced cardiac arrest, we investigated the effect of PPi on ΔΨm in adult ventricular myocytes subjected to anoxia+tachypacing. The myocytes were first subjected to tachypacing (CL = 280 ms) for 5 min before being exposed to the anoxic solution (time interval between −5 min and 0 min in Figure 8E). There were almost no changes in ΔΨm-sensitive signals both in control and PPi groups during 5 min of tachypacing alone (97.7±1.3% of baseline in control vs. 100.4±1.1% of baseline in PPi at “0 min” in Figure 8E, p>0.05). No appreciable decrease in ΔΨm was noticed during tachypacing *per se* for up to 35 minutes (data not shown). However, during the combination of tachypacing and anoxia ΔΨm-sensitive signals gradually decreased, reaching 59.5±2.9% of baseline at 20 min in control cells (n = 9) (see Figure 8A–B and Panel E). Notably, ΔΨm loss was greatly attenuated in the PPi-treated cells (n = 7) in which ΔΨm was maintained at 88.3±5.1% of baseline after 20 min of tachypacing+anoxia (see Figure 8C–D and Panel E). The effect of PPi was statistically significant between 2 and 20 min of tachypacing+anoxia. It is also worth noting that the loss of contractility was delayed in the PPi-treated myocytes as compared to control cells (contractility was maintained in 50% of PPi-treated cells vs. 22% of control cells at 20 minutes of tachypacing+anoxia, see Figure S8), although the difference did not reach statistical significance (p = 0.1096).

## Discussion

This study may be the most extensive to-date metabolic profiling of ventricular myocardium performed in a whole-heart simulation of cardiac arrest. This analysis has demonstrated that the divergence in the timing of electrical failure in the context of LDVF is related to significant inter-subject differences in metabolic state both before and during LDVF. Moreover, an unexpected finding of a large difference in the pre-ischemic level of PPi between the hearts exhibiting early and late asystole led us to a hypothesis that PPi might be able to delay ΔΨm loss during anoxia, which was confirmed in subsequent experiments using isolated cardiomyocytes.

### Heterogeneous Responses to Ischemia/metabolic Stress Despite Uniform Experimental Conditions

Our recent studies in an *ex-vivo* canine model of LDVF have shown that the time of asystole in different hearts is distributed between two distinct clusters: one around 7–10 minutes and the other close or above 20 minutes [4], [19], despite apparently homogeneous populations of dogs and uniform experimental conditions. The event of asystole was associated with an accelerated electrical depression [4], a more rapid accumulation of extracellular potassium [19], and was significantly postponed by blockade of I_K-ATP_ [4]. Thus it seemed that the dichotomy in the timing of systole was not an entirely random phenomenon, but was a result of differences in the response of myocardium to the metabolic stress imposed by LDVF, perhaps differences in some factors upstream of I_K-ATP_ activation.

Divergent responses to ischemia/metabolic stress in apparently homogeneous populations of experimental subjects have been noticed before. Vanheel at al. reported that I_K-ATP_ blockade alleviated APD shortening (presumably caused by I_K-ATP_ activation) only in *50%* of ischemic guinea pig papillary muscles [20]. O’Rourke et al. observed oscillatory opening of I_K-ATP_ due to substrate deprivation only in *44%* of ventricular myocytes [21]. In another study, brief (5 min) episodes of ischemia activated I_K-ATP_ in only *20%* of cells but in 100% of cells treated with I_K-ATP_ channel opener cromakalim [22]. Large variability of LDVF outcomes was also noted before. Wiggers et al. noted that the onset of asystole following LDVF in open-chest dogs varied between 16 and 52 minutes [23]. Unfortunately, the distribution within this range was not reported. Finally, in an open-chest rat model of VF-induced cardiac arrest deliberate I_K-ATP_ activation caused spontaneous VF termination in *approximately 50%* of cases, whereas it never occurred in the *control* group where the level of I_K-ATP_ activation was presumably smaller [24]. All these facts indicate that the electrical response of the myocardium to ischemia or LDVF, in particular in terms of I_K-ATP_ activation, may be markedly heterogeneous among hearts subjected to identical experimental conditions. The possibility that this heterogeneity is due to individual differences in metabolic profile has not been previously addressed.

We perceived the large divergence of outcomes in our model of LDVF as an excellent opportunity to explore the inter-subject differences in metabolic profile which determine one of the major end-points of cardiac arrest, the electrical failure. While we admit that our model lacks many features of more pre-clinical models of cardiac arrest which permit analysis of survival in terms of post-resuscitation cardiac function and hemodynamics in the whole organism [25], we believe that the emphasis on the inter-subject differences underlying myocardial response to metabolic stress constitutes a unique strength of our study and is highly relevant in the emerging era of personalized medicine. Indeed, ischemia or metabolic stress may be one of those critical situations where hidden vulnerabilities are revealed or enhanced, ultimately determining the ability of the individual to withstand the challenge. Understanding how the differences in metabolic state modulate individual responses to various ischemic and other critical cardiac conditions may eventually help to identify patients at risk for more adverse outcomes and possibly devise therapies tailored to patients with specific metabolic “phenotypes”.

### Lactate

Among the metabolites measured in this study, only the level of lactate was significantly higher in early asystole hearts in the time frame when early asystole occurred (5–10 minutes of LDVF). There are several possibilities that could link lactate accumulation and electrical depression during LDVF.

#### Inhibition of anaerobic glycolysis

Given that the accumulation of lactate leads to feedback inhibition of glycolysis [26], it could be possible that early-asystole hearts experienced severe glycolysis inhibition immediately before asystole occurred. This in turn could cause an acute activation of I_K-ATP_ since glycolytically-derived ATP was shown to predominantly regulate this channel [27], presumably due to a physical association between the channel subunits and enzymes of the glycolytic complex [28]. Indeed, following a rapid increase of lactate between 0 and 5 min, the rate of lactate accumulation slowed down between 5 and 10 min in early-asystole hearts (Figure 5E). Moreover, the rate of lactate accumulation between 5 and 10 min was lower in early-asystole than in late-asystole hearts, but the difference did not reach a statistical significance (1.6±0.6 vs. 3.0±0.8 µmol/g/min, p>0.05). Note however, that the actual level of ATP was not different between early- and late-asystole hearts at 5 and 10 min of LDVF (Figure 5D) and was also quite high (∼4 mM). This is clearly much above submicromolar concentrations which fully block I_K-ATP_ in excised patch experiments [29]. It is often noted that ATP levels determined in tissues, represent the cell average and thus cannot resolve heterogeneous distribution of ATP between different cellular compartments. However, assuming that in the absence of oxygen anaerobic glycolysis is the predominant source of ATP and the mitochondria become an ATP sink [30], [ATP] *at the source* should be greater or equal to the cell-averaged values. If this is true, then [ATP] visible to I_K-ATP_ is probably too high to explain early asystole in this model in terms of inhibition of anaerobic glycolysis.

#### Direct modulation of I_K-ATP_ by lactate and/or H^+^

The direct effect of lactate on I_K-ATP_ at concentrations 20–40 mM has been demonstrated in isolated ventricular myocytes and inside-out patches, even in the presence of 2–5 mM ATP [31]. Note that at 5 min of LDVF the estimated lactate concentration in early-asystole hearts was close to 13.9±1.0 mM (versus 8.4±0.7 mM in late-asystole hearts) while [ATP] in both groups was ∼4 mM. These conditions are not far from those at which the direct blocking effect of lactate on I_K-ATP_ was demonstrated in cellular models. It was also shown that a decrease in intracellular pH (a consequence of lactate production) enhances I_K-ATP_ activation [32], [33]. Moreover, intracellular acidosis may directly activate I_K-ATP_ even in the presence of physiological concentrations of ATP via a direct action of protons on the Kir6 subunit [33]. Taken together, these observations suggest that the activation effect of I_KATP_ by lactate and/or low pH may be the primary mechanism determining the outcome of LDVF in this model.

### What Determines the Rate of Lactate Accumulation during the Early Phase of LDVF?

In the absence of substrate manipulations, early-asystole hearts were characterized by a large increase in glucose utilization during the first 2 min of LDVF which coincided with a rapid increase of lactate (Figure 3). Moreover, the deliberate increase of glucose availability and uptake prior to LDVF in the *3x-glucose* group augmented lactate accumulation and promoted asystole (Figure S5). Thus, it could be possible that glucose transport via the insulin-dependent glucose transporter (GLUT4) might be involved in the high content of glucose in early asystole hearts. However, in the two early-asystole hearts from *glu-suppl* group enhanced lactate production was associated with utilization of other glycolytic metabolites (Figure S7). There was also a trend in early-asystole hearts having higher glycogen utilization in the first 5 min of LDVF (Figure 5F), which however did not reach statistical significance.

While “force-feeding” with glucose somewhat shifted the balance towards earlier asystole, the intrinsic stimulus for anaerobic glycolysis could have been related to apparent energy imbalance occurring very early in the course of LDVF. Recall that *control*-*early-asys* hearts exhibited lower levels of PCr and higher levels of ADP at 0.3 min of LDVF (Figure 2B–C). We did not find significant differences in the levels of HEP and ADP in the pre-LDVF samples (see Figure 6). Thus, it is likely that the changes in the levels of PCr and ADP in *control*-*early-asys* group occurred as an immediate response to the combination of ischemia and VF, implying that *control-early-asys* hearts had lower tolerance to this kind of stress than *control-late-asys* hearts.

### Protective Role of PPi

Among 67 metabolites analyzed in the pre-LDVF hearts, the most remarkable difference was in the level of PPi, which was 24.9 times higher in late-asys than in early-asys hearts (Figure 6). PPi is formed as a result of hydrolysis of ATP(GTP) into AMP(GMP), which occurs in many important reactions, including a priming step for fatty acid ß-oxidation and synthesis of nucleic acids, cAMP and cGMP. PPi is hydrolyzed into two molecules of inorganic phosphate by the enzyme pyrophosphatase predominantly located in mitochondrial matrix [34]. It was shown that an increase in intra-mitochondrial PPi leads to an increase in matrix volume presumably due to electrogenic K^+^ entry into mitochondria via adenine nucleotide translocase [34]. Moderate mitochondrial swelling was shown to increase respiration and fatty acid oxidation in mitochondria [35].

Mansurova et al. demonstrated that PPi can be formed in isolated mitochondria and that this process requires functional electron transport system [18]. Furthermore, there is evidence suggesting that the high-energy bond in PPi can be utilized for ATP synthesis and maintenance of ΔΨm, both processes presumably mediated by a proton-pumping, inner-membrane-bound enzyme pyrophosphate dehydrogenase [10], [36]. Given that a large fraction of glycolytic ATP is hydrolyzed by F_1_F_0_ -ATPase in an attempt to maintain ΔΨm after oxidative phosphorylation is halted [30], it is plausible that elevated level of PPi prior to ischemia could protect against early asystole by decreasing the rate of mitochondrial ATP hydrolysis and thus preventing over-stimulation of anaerobic glycolysis and excessive accumulation of lactate. In support of this hypothesis, our study in cultured myocytes demonstrated that PPi prevents ΔΨm depolarization during short-term anoxia (see Figure 7). Furthermore, as a more relevant model to VF/ischemia, we tested the cardioprotective effect of PPi on maintenance of ΔΨm using rabbit adult ventricular myocytes subjected to anoxia and tachypacing. The PPi-treated myocytes were able to maintain ΔΨm much better than untreated cells throughout 20 min of anoxia+tachypacing (see Figure 8). There was also a trend towards a better maintenance of contractility in the PPi-treated myocytes as compared to control cells, albeit not statistically significant (see Figure S8). Thus, it is plausible that the high level of PPi in the preischemic late-asystole hearts postpones the time of asystole by delaying ΔΨm depolarization in the course of VF/ischemia, which presumably reduces the demand for glycolytic ATP under metabolic stress.

### Link between Metabolic State and the Incidence of Asystole

**Figure 9:**
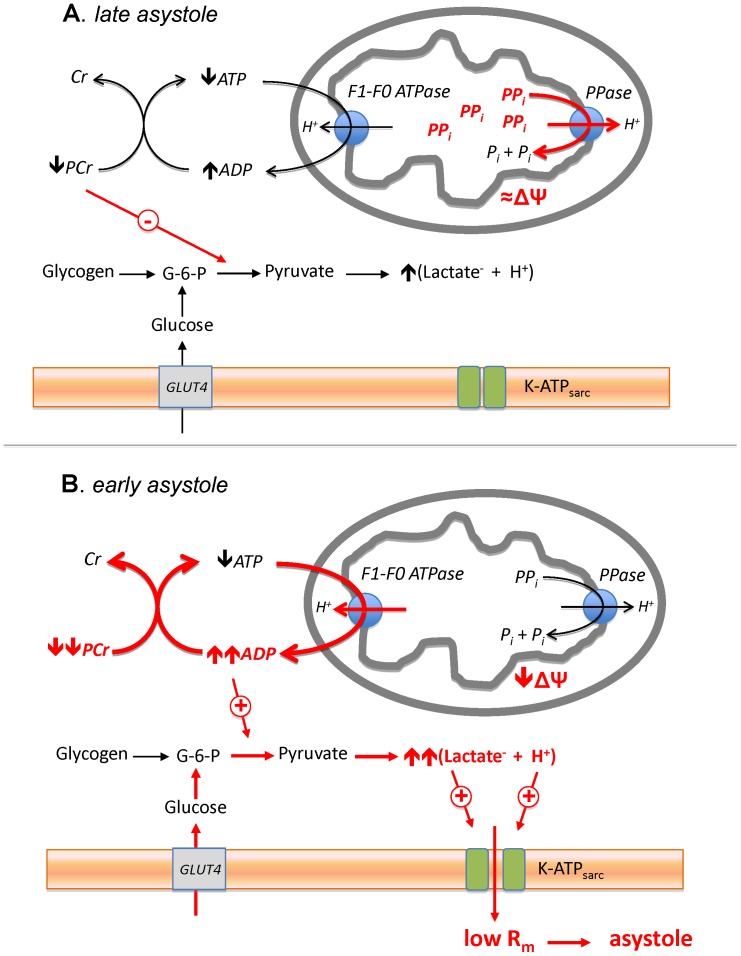
The proposed mechanism whereby PPi protects against early electrical failure in the course of LDVF. Red color highlights the important differences between the early-asystole (**A**) and late-asystole (**B**) hearts, either observed or postulated. The pre-LDVF *late-asystole* heart contains high level of PPi, which contributes to the maintenance of ΔΨ via proton-pumping pyrophosphatase (*PPase*) and thus limits ATP hydrolysis by F1–F0 ATPase and the rise in ADP concentration at the early stage of VF/ischemia. This in turn conserves PCr, which exerts inhibitory influence on some steps of anaerobic glycolysis. In contrast, in *early-asystole* heart containing a small amount of PPi, a large fraction of glycolytic ATP is hydrolyzed by F_1_F_0_ -ATPase in an attempt to maintain ΔΨm after oxidative phosphorylation is halted. A decrease in [ATP] is partially compensated at the expense of PCr, but nevertheless the net result is an increase in [ADP] and a decrease in [PCr]. These changes lead to stimulation of anaerobic glycolysis beyond the level occurring in *late-asystole* heart and result in accumulation of excessive amounts of lactate and protons. This in turn activates sarcolemmal K-ATP channel (K-ATP_sarc_) which decreases the membrane resistance (R_m_) to the level sufficient to cause “metabolic sink block” [37] and full inexcitability (asystole). G-6-P, glucose-6-phosphate.

Summarizing this work, we would like to propose a speculative mechanism linking metabolic and electrophysiological phenomena observed in our study (Figure 9). For reasons yet to be determined, in our experimental model some isolated hearts contain much more PPi than other hearts. We surmise that PPi in canine hearts contributes to the maintenance of ΔΨ at the early stage of LDVF similar to the effect of PPi observed during anoxia in neonatal and adult cardiomyocytes (Figures 7 and 8), and thus limits mitochondrial ATP hydrolysis. This effect conserves energy charge within the first few minutes of LDVF in late-asystole hearts, and consequently limits activation of anaerobic glycolysis and subsequent accumulation of lactate. Conversely, hearts with low amount of PPi (early-asystole hearts) lack this protective mechanism and produce excessive amounts of lactate and protons, which in turn activate I_K-ATP_ to the level sufficient to cause “metabolic sink block” [37] and full inexcitability. Mechanisms regulating the level of PPi in myocardium remain to be elucidated but definitely warrant further investigation given the cardioprotective potential of this small metabolite demonstrated in this study.

### Limitations

Heart isolation and perfusion in the Langendorff apparatus most likely affects the metabolic profile at baseline and during ischemia [6] and thus may influence the outcomes of this study. This is a common limitation of isolated heart models and is a price to pay for the greater degree of experimental control. A study is under way in our laboratory to investigate in detail the metabolic differences between *in-situ* hearts and isolated hearts perfused with blood or crystalline solution. In any event, the metabolic and electrophysiological differences observed between early-asystole and late-asystole hearts occurred under the same experimental conditions and therefore represented intrinsic inter-subject differences.

In this study the effect of PPi on ΔΨ under metabolic stress was demonstrated using neonatal and adult ventricular myocytes. However, it remains to be elucidated whether the similar effect of PPi on ΔΨ during VF/ischemia can be seen in intact hearts.

Using isolated, retrogradely perfused hearts and taking multiple tissue samples during ischemia precluded us from modeling and analysis of electrophysiological and metabolic dynamics associated with cardio-pulmonary resuscitation and return of spontaneous circulation. A differently designed future study should address these issues.

## Supporting Information

Figure S1 **Typical examples of electrical activity following LDVF termination.** **A.** An example of VF termination followed by ventricular tachycardia (VT) and subsequent asystole interrupted only by a short burst of bradycardia occurring during tissue sampling and most likely attributable to the acute injury cause by cutting the tissue. **B.** An example of VF termination followed by a period of silence lasting slightly over one minute and subsequent bradycardia lasting for another minute. After that no activity was observed. **C.** an example of VF termination followed by a period of silence over 20 seconds and subsequent bradycardia <20 bpm. Oblique white arrowheads indicate artifacts caused by tissue sampling.
(TIF)

Figure S2 **The levels of total pool of adenine nucleotides (ΣAd = ATP+ADP+AMP) during LDVF.** **A.** *Control-late-asys* group. **B.** *Control-early-asys* group. ΣAd gradually decreased during LDVF. Together with the continuous increase in the level of inosine (see Figure 2F in the main text), these results indicate the adenine nucleotide degradation pathway was activated under these conditions. However, there were no statistical differences in the levels of ΣAd at any time points studied. The other intermediates of adenine nucleotide degradation pathways (i.e. adenosine, hypoxanthine, xanthine) were not detected by GC/MS or HPLC.
(TIF)

Figure S3 **Changes in the levels of NAD and NADH during LDVF.** **A.** Total pool of the oxidized and reduced forms (NADtotal: NAD^+^+NADH). **B.** NADH. There were no statistical differences in the levels of NADtotal and NADH between the *control-late-asys* and *control-early-asys* groups at any time points studied.
(TIF)

Figure S4 **Changes in the TCA cycle intermediates, related amino acids and free fatty acids during LDVF.** The data are compared between control-early-asys and control-late-asys hearts. The TCA cycle data are the same as in Figure 4 in the main text. Values are indicated as arbitrary units (y-axis). For clarity, the data are presented as bar graphs, and x-axis indicates the approximate time of ischemia (min). Among five TCA intermediates that were detected by GC/MS in this study, the level of citrate at 2 min was significantly different between control-early-asys and control-late-asys hearts (asterisk) The transient increase in the level of citrate in control-early-asys hearts could be attributed to the catabolism from fatty acids and/or branched-chain amino acid, which are two possible sources of acetyl-CoA. However, none of fatty acids and branched amino acids that could be measured by GC/MS showed a consistent decrease during LDVF, and there were not statistically differences between control-early-asys and control-late-asys hearts. Therefore, the most likely source of citrate increase in control-early-asys hearts was acetyl-CoA from pyruvate, which would be consistent with the evidence of enhanced anaerobic glycolysis (see Figure 3 in the main text). Note that the level of succinate increased throughout 20 min of LDVF, coupled with parallel increase in alanine during LDVF, which indicates the presence of active anaplerotic process by which α-ketoglutarate is formed via transamination of some amino acids [38]. This reaction provides an input for anaerobic GTP formation in the TCA cycle, which may contribute to the energy preservation in mitochondria during LDVF. However, the levels of succinate and alanine were not significantly different between control-early-asys and control-late-asys hearts throughout 20 min of ischemia.
(TIF)

Figure S5 **Effects of switching to perfusate with high concentration of glucose prior to LDVF.** The tissue contents of glucose (**A**), lactate (**B**) and glycogen (**C**) were compared between *control* group (5.5 mmol/L glucose, solid line) and *3x-glucose* group (16.5 mmol/L glucose, dotted line). (*:p<0.05) **D.** Cumulative probability of asystole during 20 min of LDVF in *control* and *3x-glucose* hearts (p<0.05). Note that enhanced glucose delivery to the myocardium accelerates asystole overall, yet there is still a notion of two clusters in *3x-glucose* group: early asystole occurring before 8 min of ischemia and late asystole occurring after 16 min of ischemia.
(TIF)

Figure S6 **Effects of the glucose supplementation protocol.** The glycogen content (**A**), the lactate level (**B**), the glucose level (**C**), and VF maintenance probability (**D**) are compared between *control* group (solid line) and *glu-suppl* group (dashed line). Note that in Panel D the asystole probability data is derived from *glu-suppl* (n = 4) and *glu-suppl-ex* (n = 7) groups combined together. Glucose supplementation protocol significantly increased the pre-ischemic level of glycogen, but did not alter the amount of glycogen utilized during 20 min of ischemia (see inset in Panel A). Also, the average levels of glucose and lactate were very similar in *glu-suppl* and *control* group at all time points analyzed. Lastly, the glucose supplementation protocol did not significantly alter the probability of asystole (p>0.05), although there is a slight trend of earlier asystole in *glu-suppl* group.
(TIF)

Figure S7 **Data from individual** ***glu-suppl*** **hearts (n = 4) emphasizing the bimodal response to LDVF. A.** Asystole time clearly showing division into late and early asystole. Two hearts underwent asystole during the first 10 min (red) while other two hearts maintained electrical activity throughout 20 min of LDVF (blue). **B.** The two *early-asys* hearts increased lactate significantly faster than the two *late-asys* hearts. **C.** Unlike in the *control* group (see Figure 3 in the main text), in *glu-suppl* group the glucose content at the onset of LDVF was not different between *early-* and *late-asys* hearts. Note however that the two *early-asys* hearts in *glu-suppl* group exhibited high initial levels of other glycolytic substrates or intermediates followed by a large decrease in the level of these compounds during the first 2 min of LDVF. One *early-asys* heart (Δ) contained a high level of fructose at the onset, followed by a large reduction during the first 2 min of LDVF (**D**)**.** The other *early-asys* heart (x) contained high levels of two glycolytic intermediates (2-phosphoglycerol and 3-phosphoglycerol) at the onset of LDVF, followed by a large reduction in the levels of these compounds during the first 2 min of LDVF (**E–F**). From Figures S6 and S7 we conclude that the glucose supplementation protocol did increase the level of glycogen, but did not change the bimodal outcome of LDVF and the association between early asystole and a high level of lactate accumulation observed also in the *control* group.
(TIF)

Figure S8 **Effect of PPi on maintenance of contractility during simulated-demand ischemia in adult myocytes.** Cellular contractility during anoxia+tachypacing was maintained longer in the PPi-treated cells (red line) than in control cells (blue line) although the difference did not reach statistical significance by log-rank test. Yet it should be noted that at 20 min of anoxia+tachypacing, only 22% of control myocytes maintained contractility, while in PPi group 50% of myocytes maintained contractility at this time point.
(TIF)

Table S1 **Tissue sampling time.**
(DOC)

Table S2 **Animal profile and preparation time for hearts in** ***control*** **group.**
(DOC)

Text S1 **Detailed methods.**
(DOC)

## References

[1] Hallstrom A, Herlitz J, Kajino K, Olasveengen TM (2009) Treatment of asystole and PEA. Resuscitation 80: 975–976. 19581035 10.1016/j.resuscitation.2009.05.019

[2] Cobb LA, Fahrenbruch CE, Olsufka M, Copass MK (2002) Changing incidence of out-of-hospital ventricular fibrillation, 1980–2000. JAMA 288: 3008–3013. 12479765 10.1001/jama.288.23.3008

[3] Gang UJ, Jons C, Jorgensen RM, Abildstrom SZ, Haarbo J, et al (2010) Heart rhythm at the time of death documented by an implantable loop recorder. Europace 12(2): 254–60. 20019013 10.1093/europace/eup383

[4] Taylor TG, Venable PW, Shibayama J, Warren M, Zaitsev AV (2012) Role of KATP channel in electrical depression and asystole during long-duration ventricular fibrillation in ex vivo canine heart. Am J Physiol Heart Circ Physiol 302(11): H2396–2409. 22467302 10.1152/ajpheart.00752.2011 PMC3378304

[5] Cross HR, Opie LH, Radda GK, Clarke K (1996) Is a high glycogen content beneficial or detrimental to the ischemic rat heart? A controversy resolved. Circ Res 78: 482–491. 8593707 10.1161/01.res.78.3.482

[6] Jennings RB, Reimer KA, Hill ML, Mayer SE (1981) Total ischemia in dog hearts, in vitro. 1. Comparison of high energy phosphate production, utilization, and depletion, and of adenine nucleotide catabolism in total ischemia in vitro vs. Severe ischemia in vivo. Circ Res 49: 892–900. 7273360 10.1161/01.res.49.4.892

[7] Worley SJ, Swain JL, Colavita PG, Smith WM, Ideker RE (1985) Development of an endocardial-epicardial gradient of activation rate during electrically induced, sustained ventricular fibrillation in dogs. Am J Cardiol 55: 813–820. 3976529 10.1016/0002-9149(85)90162-6

[8] Kusuoka H, Chacko VP, Marban E (1992) Myocardial energetics during ventricular fibrillation investigated by magnetization transfer nuclear magnetic resonance spectroscopy. Circ Res 71: 1111–1122. 1394873 10.1161/01.res.71.5.1111

[9] Baker M (2011) Metabolomics: From small molecules to big ideas. Nat Med 8: 117–121.

[10] Pereira-da-Silva L, Sherman M, Lundin M, Baltscheffsky H (1993) Inorganic pyrophosphate gives a membrane potential in yeast mitochondria, as measured with the permeant cation tetraphenylphosphonium. Arch Biochem Biophys 304(2): 310–3. 8394052 10.1006/abbi.1993.1355

[11] Venable PW, Taylor TG, Shibayama J, Warren M, Zaitsev AV (2010) Complex structure of electrophysiological gradients emerging during long-duration ventricular fibrillation in the canine heart. Am J Physiol Heart Circ Physiol 299: H1405–1418. 20802138 10.1152/ajpheart.00419.2010 PMC2993199

[12] Volonte MG, Yuln G, Quiroga P, Consolini AE (2004) Development of an HPLC method for determination of metabolic compounds in myocardial tissue. J Pharm Biomed Anal 35: 647–653. 15137992 10.1016/j.jpba.2004.02.002

[13] Shakoury-Elizeh M, Protchenko O, Berger A, Cox J, Gable K, et al (2010) Metabolic response to iron deficiency in saccharomyces cerevisiae. J Biol Chem 285: 14823–14833. 20231268 10.1074/jbc.M109.091710 PMC2863190

[14] Passonneau JV, Lauderdale VR (1974) A comparison of three methods of glycogen measurement in tissues. Anal Biochem 60: 405–412. 4844560 10.1016/0003-2697(74)90248-6

[15] Zaniboni M, Pollard AE, Yang L, Spitzer KW (2000) Beat-to-beat repolarization variability in ventricular myocytes and its suppression by electrical coupling. Am J Physiol Heart Circ Physiol 278: H677–687. 10710334 10.1152/ajpheart.2000.278.3.H677

[16] Warren M, Spitzer KW, Steadman BW, Rees TD, Venable P, et al (2010) High-precision recording of the action potential in isolated cardiomyocytes using the near-infrared fluorescent dye di-4-anbdqbs. Am J Physiol Heart Circ Physiol 299: H1271–1281. 20601458 10.1152/ajpheart.00248.2010 PMC2957348

[17] Jennings RB, Steenbergen C Jr (1985) Nucleotide metabolism and cellular damage in myocardial ischemia. Annu Rev Physiol 47: 727–49. 2581508 10.1146/annurev.ph.47.030185.003455

[18] Mansurova SE, Shakhov YA, Belyakova TN, Kulaev IS (1975) Synthesis of inorganic pyrophosphate by animal tissue mitochondria. FEBS Lett 55: 94–98. 166891 10.1016/0014-5793(75)80967-7

[19] Taylor TG, Venable PW, Shibayama J, Warren M, Booth A, et al (2011) Role of hyperkalemia in the transmural activation rate gradient and asystole during long-duration ventricular fibrillation (LDVF) in isolated canine heart. Circulation 124: A9 (Abstract)..

[20] Vanheel B, de Hemptinne A (1992) Influence of K-ATP channel modulation on net potassium efflux from ischaemic mammalian cardiac tissue. Cardiovasc Res 26: 1030–1039. 1291079 10.1093/cvr/26.11.1030

[21] O’Rourke B, Ramza BM, Marban E (1994) Oscillations of membrane current and excitability driven by metabolic oscillations in heart cells. Science 265: 962–966. 8052856 10.1126/science.8052856

[22] Henry P, Popescu A, Puceat M, Hinescu ME, Escande D (1996) Acute simulated ischaemia produces both inhibition and activation of K^+^ currents in isolated ventricular myocytes. Cardiovasc Res 32: 930–939. 8944824

[23] Wiggers CJ, Bell JR, Paine M (1930) Studies of ventricular fibrillation caused by electric shock: II. Cinematographic and electrocardiographic observations of the natural process in the dog’s heart. Its inhibition by potassium and the revival of coordinated beats by calcium. Am Heart J 5: 351–365. 10.1046/j.1542-474X.2003.08316.x PMC6932455 14510663

[24] Tang W, Weil MH, Sun S, Pernat A, Mason E (2000) K(ATP) channel activation reduces the severity of postresuscitation myocardial dysfunction. Am J Physiol Heart Circ Physiol 279: H1609–1615. 11009447 10.1152/ajpheart.2000.279.4.H1609

[25] Ayoub IM, Kolarova JD, Kantola RL, Radhakrishnan J, Wang S, et al (2007) Gazmuri RJ. Zoniporide preserves left ventricular compliance during ventricular fibrillation and minimizes postresuscitation myocardial dysfunction through benefits on energy metabolism. Crit Care Med 10: 2329–36. 10.1097/01.ccm.0000280569.87413.74 17944021

[26] Rovetto MJ, Lamberton WF, Neely JR (1975) Mechanisms of glycolytic inhibition in ischemic rat hearts. Circ Res 37: 742–751. 157 10.1161/01.res.37.6.742

[27] Weiss JN, Lamp ST (1987) Glycolysis preferentially inhibits ATP-sensitive K^+^ channels in isolated guinea pig cardiac myocytes. Science 238: 67–69. 2443972 10.1126/science.2443972

[28] Hong M, Kefaloyianni E, Bao L, Malester B, Delaroche D, et al (2011) Cardiac ATP-sensitive K^+^ channel associates with the glycolytic enzyme complex. FASEB J 25: 2456–2467. 21482559 10.1096/fj.10-176669 PMC3114533

[29] Noma A (1983) ATP-regulated K^+^ channels in cardiac muscle. Nature 305: 147–148. 6310409 10.1038/305147a0

[30] Ganitkevich V, Mattea V, Benndorf K (2010) Glycolytic oscillations in single ischemic cardiomyocytes at near anoxia. J Gen Physiol 135: 307–319. 20231372 10.1085/jgp.200910332 PMC2847920

[31] Keung EC, Li Q (1991) Lactate activates ATP-sensitive potassium channels in guinea pig ventricular myocytes. J Clin Invest 88: 1772–1777. 1939661 10.1172/JCI115497 PMC295726

[32] Koyano T, Kakei M, Nakashima H, Yoshinaga M, Matsuoka T, et al (1993) ATP-regulated K^+^ channels are modulated by intracellular H^+^ in guinea-pig ventricular cells. J Physiol 463: 747–766. 8246204 10.1113/jphysiol.1993.sp019620 PMC1175369

[33] Xu H, Cui N, Yang Z, Wu J, Giwa LR, et al (2001) Direct activation of cloned K(ATP) channels by intracellular acidosis. J Biol Chem 276: 12898–12902. 11278532 10.1074/jbc.M009631200

[34] Davidson AM, Halestrap AP (1989) Inhibition of mitochondrial-matrix inorganic pyrophosphatase by physiological [Ca^2+^], and its role in the hormonal regulation of mitochondrial matrix volume. Biochem J 258: 817–821. 2543362 10.1042/bj2580817 PMC1138437

[35] Halestrap AP, Dunlop JL (1986) Intramitochondrial regulation of fatty acid beta-oxidation occurs between flavoprotein and ubiquinone. A role for changes in the matrix volume. Biochem J 239: 559–565. 3827814 10.1042/bj2390559 PMC1147323

[36] Yi YJ, Sutovsky M, Kennedy C, Sutovsky P (2012) Identification of the inorganic pyrophosphate metabolizing, atp substituting pathway in mammalian spermatozoa. PloS one 7: e34524. 22485177 10.1371/journal.pone.0034524 PMC3317647

[37] Akar FG, Aon MA, Tomaselli GF, O’Rourke B (2005) The mitochondrial origin of postischemic arrhythmias. J Clin Invest 12: 3527–35. 10.1172/JCI25371 PMC1280968 16284648

[38] Peuhkurinen KJ, Takala TE, Nuutinen EM, Hassinen IE (1983) Tricarboxylic acid cycle metabolites during ischemia in isolated perfused rat heart. Am J Physiol 244: H281–288. 6824095 10.1152/ajpheart.1983.244.2.H281

