# Supplementary figures and images for: Metabolic Determinants of Electrical Failure in Ex-Vivo Canine Model of Cardiac Arrest: Evidence for the Protective Role of Inorganic Pyrophosphate
Source: PLoS One. 2013 Mar 8;8(3):e57821. doi: 10.1371/journal.pone.0057821 (PMC3592894; doi:10.1371/journal.pone.0057821)

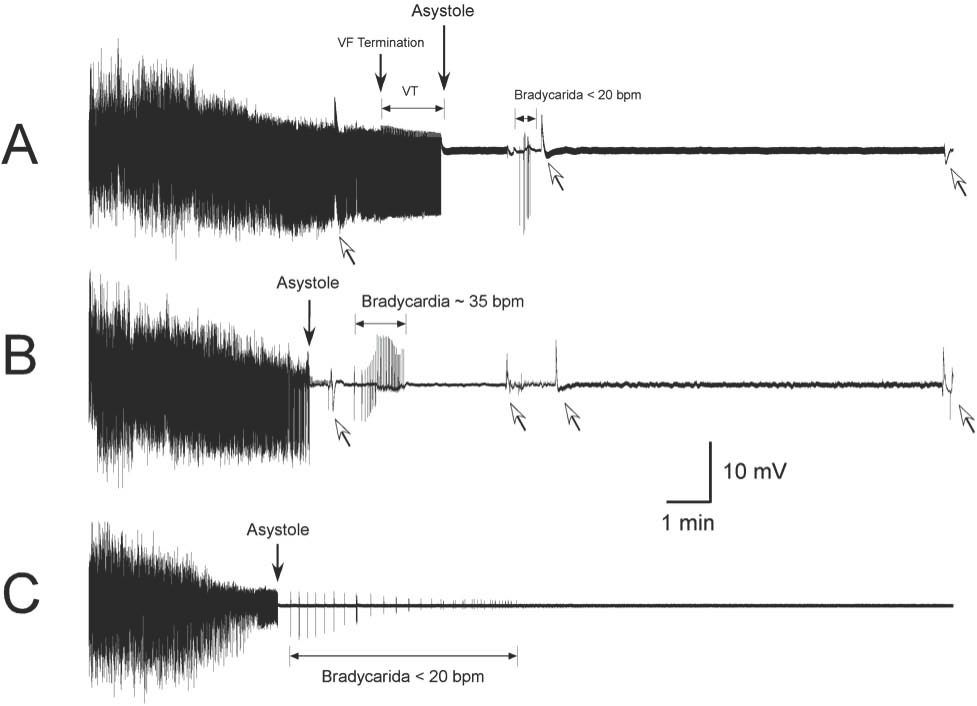

Supplement: Figure S1 — Typical examples of electrical activity following LDVF termination. A. An example of VF termination followed by ventricular tachycardia (VT) and subsequent asystole interrupted only by a short burst of bradycardia occurring during tissue sampling and most likely attributable to the acute injury cause by cutting the tissue. B. An example of VF termination followed by a period of silence lasting slightly over one minute and subsequent bradycardia lasting for another minute. After that no activity was observed. C. an example of VF termination followed by a period of silence over 20 seconds and subsequent bradycardia <20 bpm. Oblique white arrowheads indicate artifacts caused by tissue sampling. (TIF) [file pone.0057821.s001.tif]

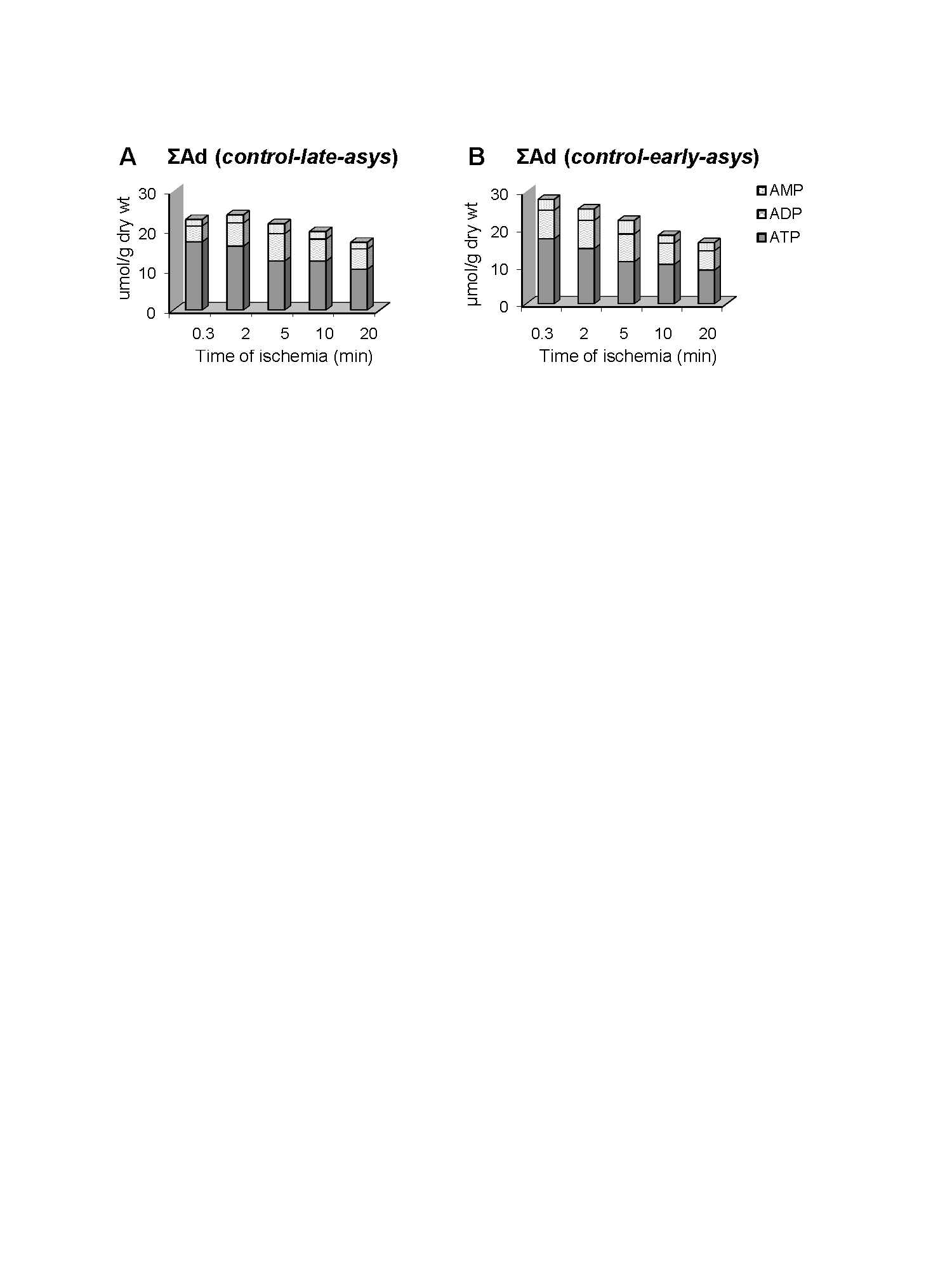

Supplement: Figure S2 — The levels of total pool of adenine nucleotides (ΣAd = ATP+ADP+AMP) during LDVF. A. Control-late-asys group. B. Control-early-asys group. ΣAd gradually decreased during LDVF. Together with the continuous increase in the level of inosine (see Figure 2F in the main text), these results indicate the adenine nucleotide degradation pathway was activated under these conditions. However, there were no statistical differences in the levels of ΣAd at any time points studied. The other intermediates of adenine nucleotide degradation pathways (i.e. adenosine, hypoxanthine, xanthine) were not detected by GC/MS or HPLC. (TIF) [file pone.0057821.s002.tif]

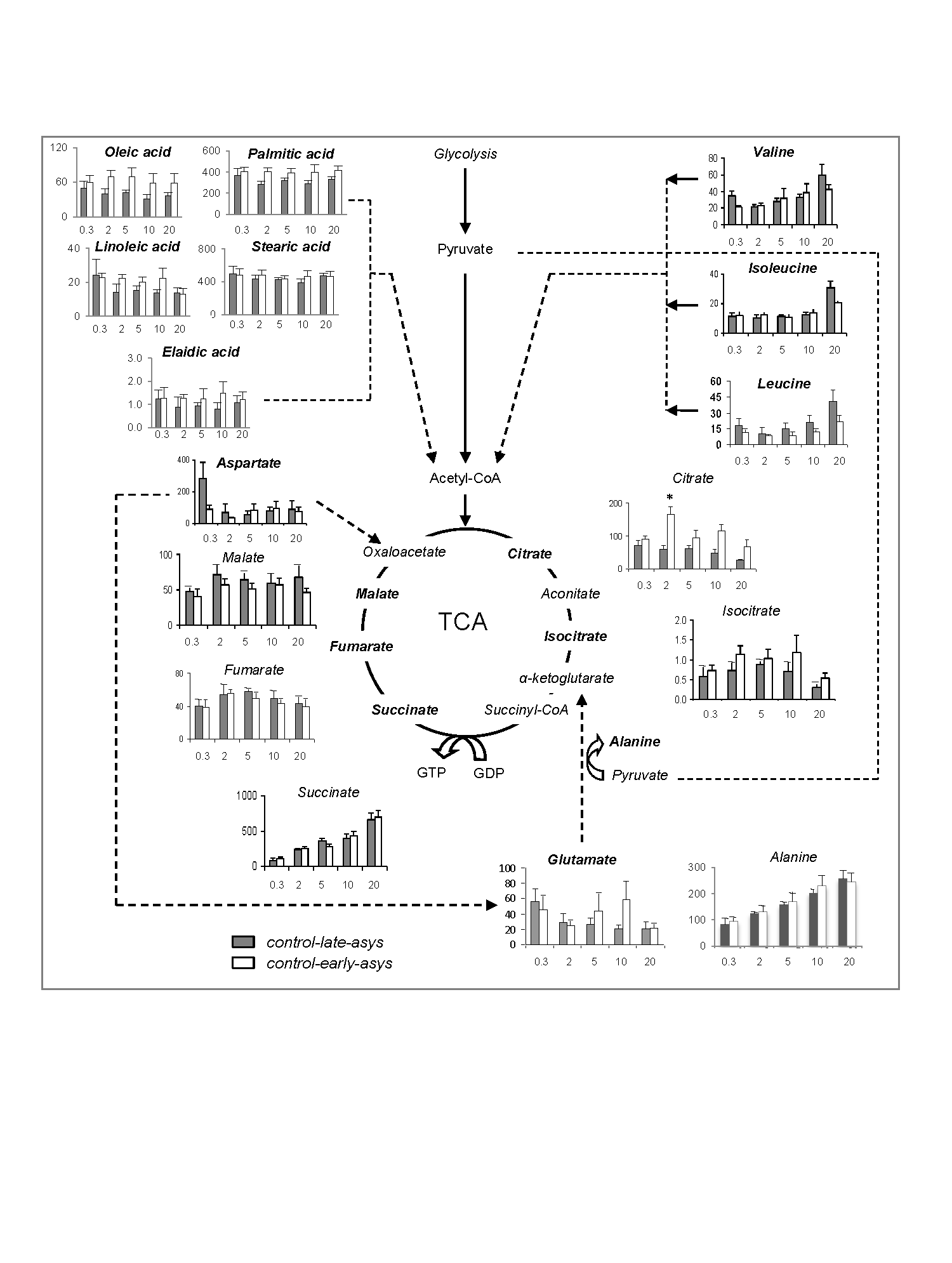

Supplement: Figure S4 — Changes in the TCA cycle intermediates, related amino acids and free fatty acids during LDVF. The data are compared between control-early-asys and control-late-asys hearts. The TCA cycle data are the same as in Figure 4 in the main text. Values are indicated as arbitrary units (y-axis). For clarity, the data are presented as bar graphs, and x-axis indicates the approximate time of ischemia (min). Among five TCA intermediates that were detected by GC/MS in this study, the level of citrate at 2 min was significantly different between control-early-asys and control-late-asys hearts (asterisk) The transient increase in the level of citrate in control-early-asys hearts could be attributed to the catabolism from fatty acids and/or branched-chain amino acid, which are two possible sources of acetyl-CoA. However, none of fatty acids and branched amino acids that could be measured by GC/MS showed a consistent decrease during LDVF, and there were not statistically differences between control-early-asys and control-late-asys hearts. Therefore, the most likely source of citrate increase in control-early-asys hearts was acetyl-CoA from pyruvate, which would be consistent with the evidence of enhanced anaerobic glycolysis (see Figure 3 in the main text). Note that the level of succinate increased throughout 20 min of LDVF, coupled with parallel increase in alanine during LDVF, which indicates the presence of active anaplerotic process by which α-ketoglutarate is formed via transamination of some amino acids [38]. This reaction provides an input for anaerobic GTP formation in the TCA cycle, which may contribute to the energy preservation in mitochondria during LDVF. However, the levels of succinate and alanine were not significantly different between control-early-asys and control-late-asys hearts throughout 20 min of ischemia. (TIF) [file pone.0057821.s004.tif]

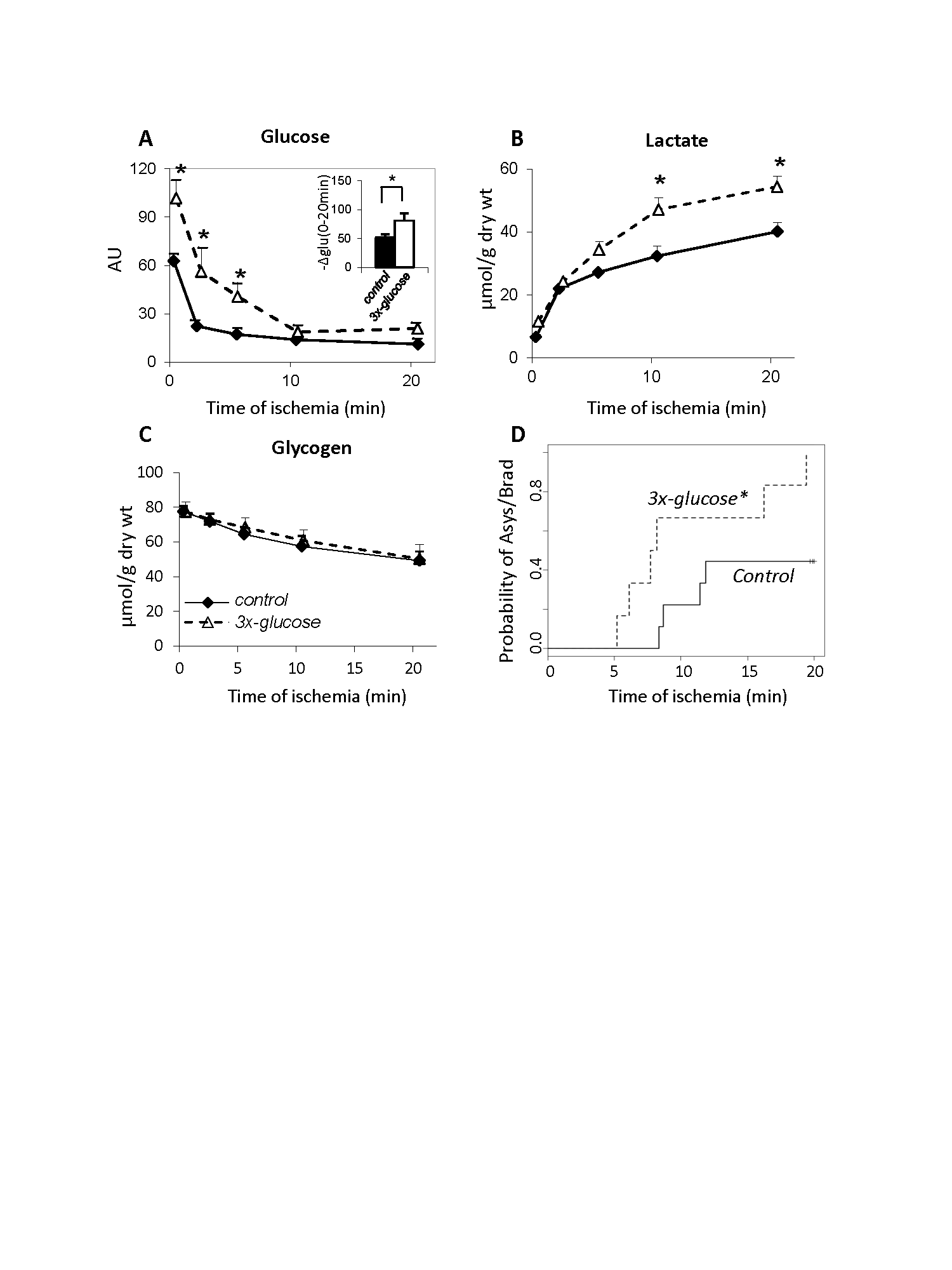

Supplement: Figure S5 — Effects of switching to perfusate with high concentration of glucose prior to LDVF. The tissue contents of glucose (A), lactate (B) and glycogen (C) were compared between control group (5.5 mmol/L glucose, solid line) and 3x-glucose group (16.5 mmol/L glucose, dotted line). (*:p<0.05) D. Cumulative probability of asystole during 20 min of LDVF in control and 3x-glucose hearts (p<0.05). Note that enhanced glucose delivery to the myocardium accelerates asystole overall, yet there is still a notion of two clusters in 3x-glucose group: early asystole occurring before 8 min of ischemia and late asystole occurring after 16 min of ischemia. (TIF) [file pone.0057821.s005.tif]

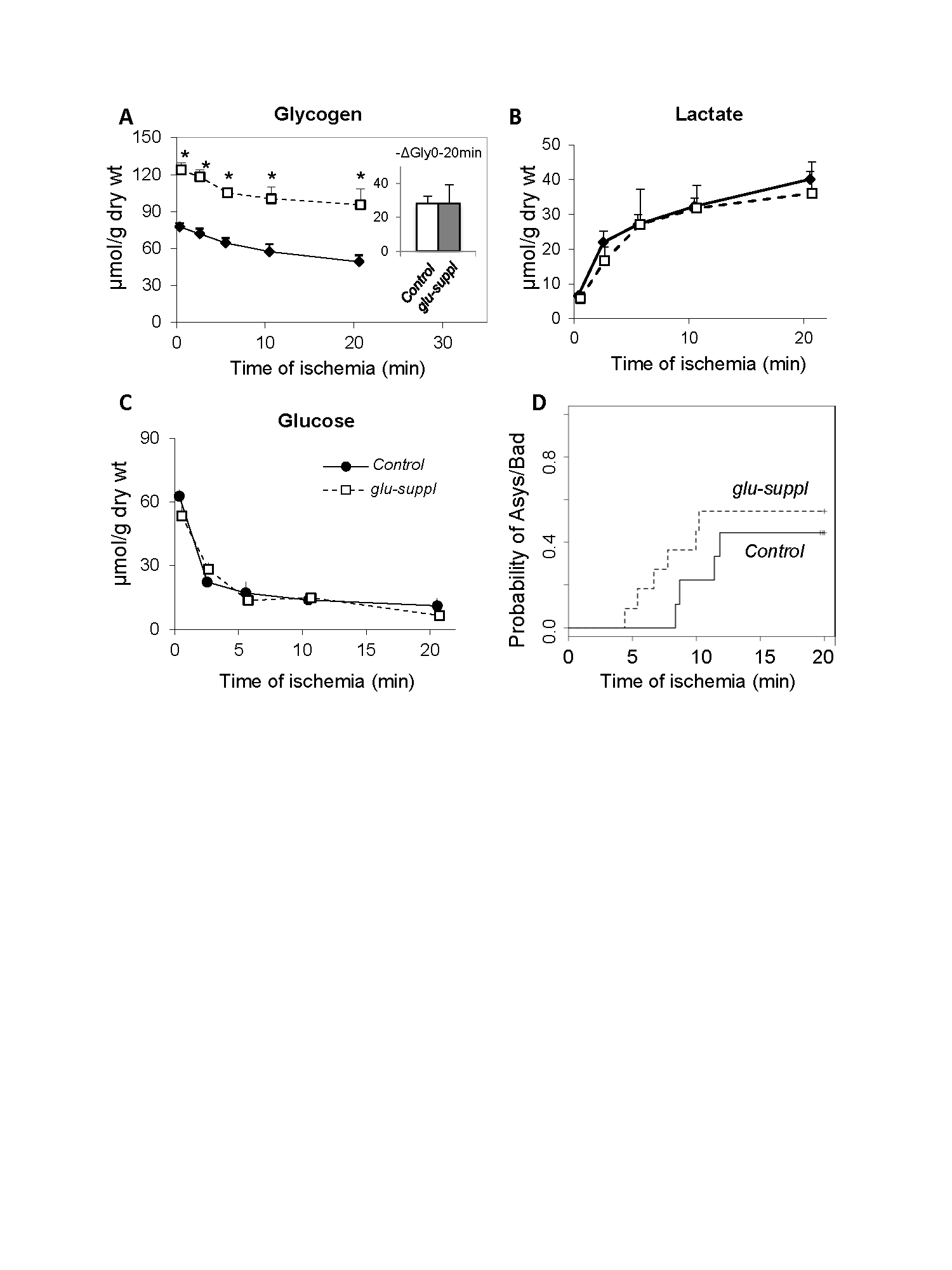

Supplement: Figure S6 — Effects of the glucose supplementation protocol. The glycogen content (A), the lactate level (B), the glucose level (C), and VF maintenance probability (D) are compared between control group (solid line) and glu-suppl group (dashed line). Note that in Panel D the asystole probability data is derived from glu-suppl (n = 4) and glu-suppl-ex (n = 7) groups combined together. Glucose supplementation protocol significantly increased the pre-ischemic level of glycogen, but did not alter the amount of glycogen utilized during 20 min of ischemia (see inset in Panel A). Also, the average levels of glucose and lactate were very similar in glu-suppl and control group at all time points analyzed. Lastly, the glucose supplementation protocol did not significantly alter the probability of asystole (p>0.05), although there is a slight trend of earlier asystole in glu-suppl group. (TIF) [file pone.0057821.s006.tif]

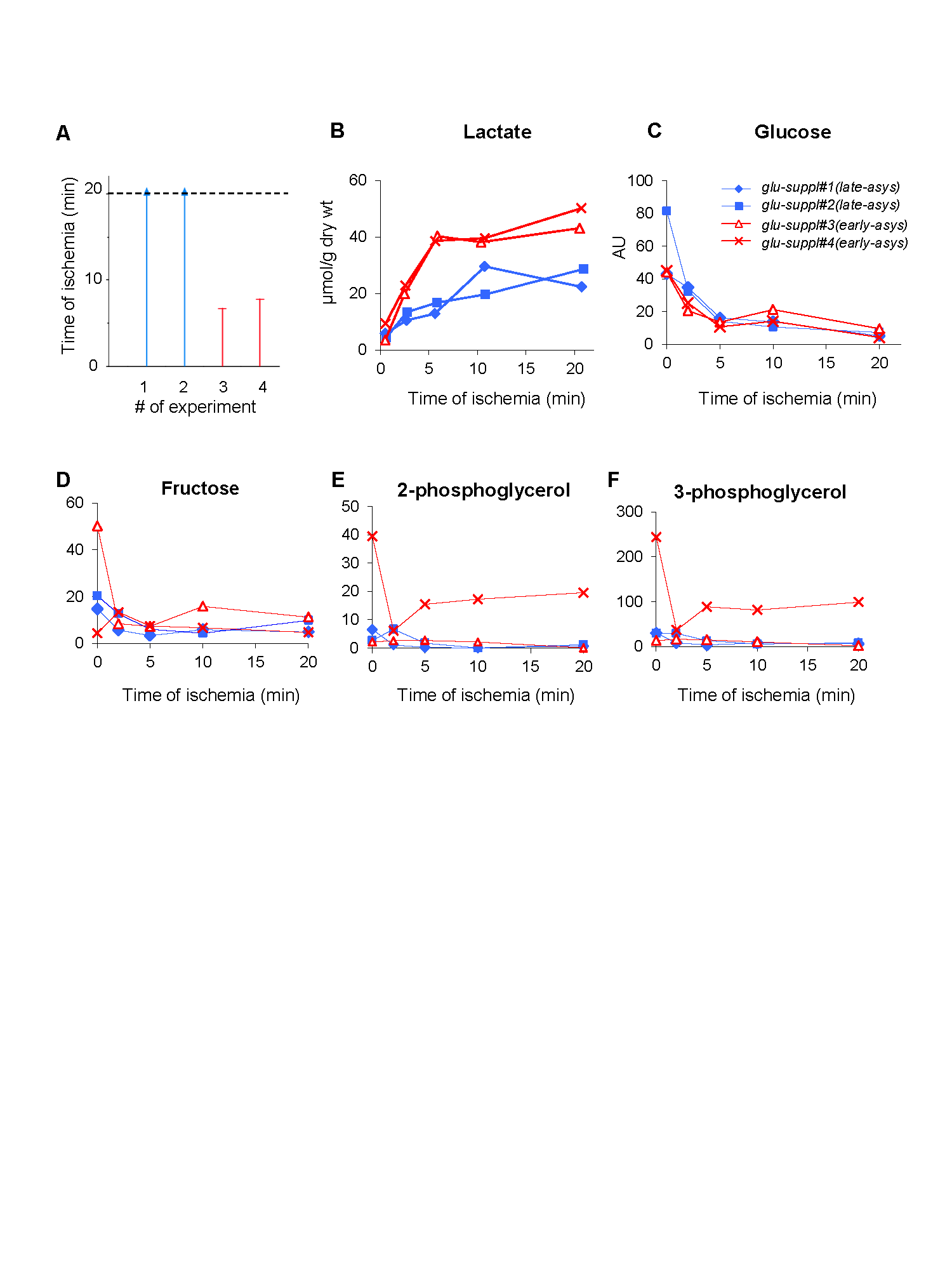

Supplement: Figure S7 — Data from individual glu-suppl hearts (n = 4) emphasizing the bimodal response to LDVF. A. Asystole time clearly showing division into late and early asystole. Two hearts underwent asystole during the first 10 min (red) while other two hearts maintained electrical activity throughout 20 min of LDVF (blue). B. The two early-asys hearts increased lactate significantly faster than the two late-asys hearts. C. Unlike in the control group (see Figure 3 in the main text), in glu-suppl group the glucose content at the onset of LDVF was not different between early- and late-asys hearts. Note however that the two early-asys hearts in glu-suppl group exhibited high initial levels of other glycolytic substrates or intermediates followed by a large decrease in the level of these compounds during the first 2 min of LDVF. One early-asys heart (Δ) contained a high level of fructose at the onset, followed by a large reduction during the first 2 min of LDVF (D). The other early-asys heart (x) contained high levels of two glycolytic intermediates (2-phosphoglycerol and 3-phosphoglycerol) at the onset of LDVF, followed by a large reduction in the levels of these compounds during the first 2 min of LDVF (E–F). From Figures S6 and S7 we conclude that the glucose supplementation protocol did increase the level of glycogen, but did not change the bimodal outcome of LDVF and the association between early asystole and a high level of lactate accumulation observed also in the control group. (TIF) [file pone.0057821.s007.tif]

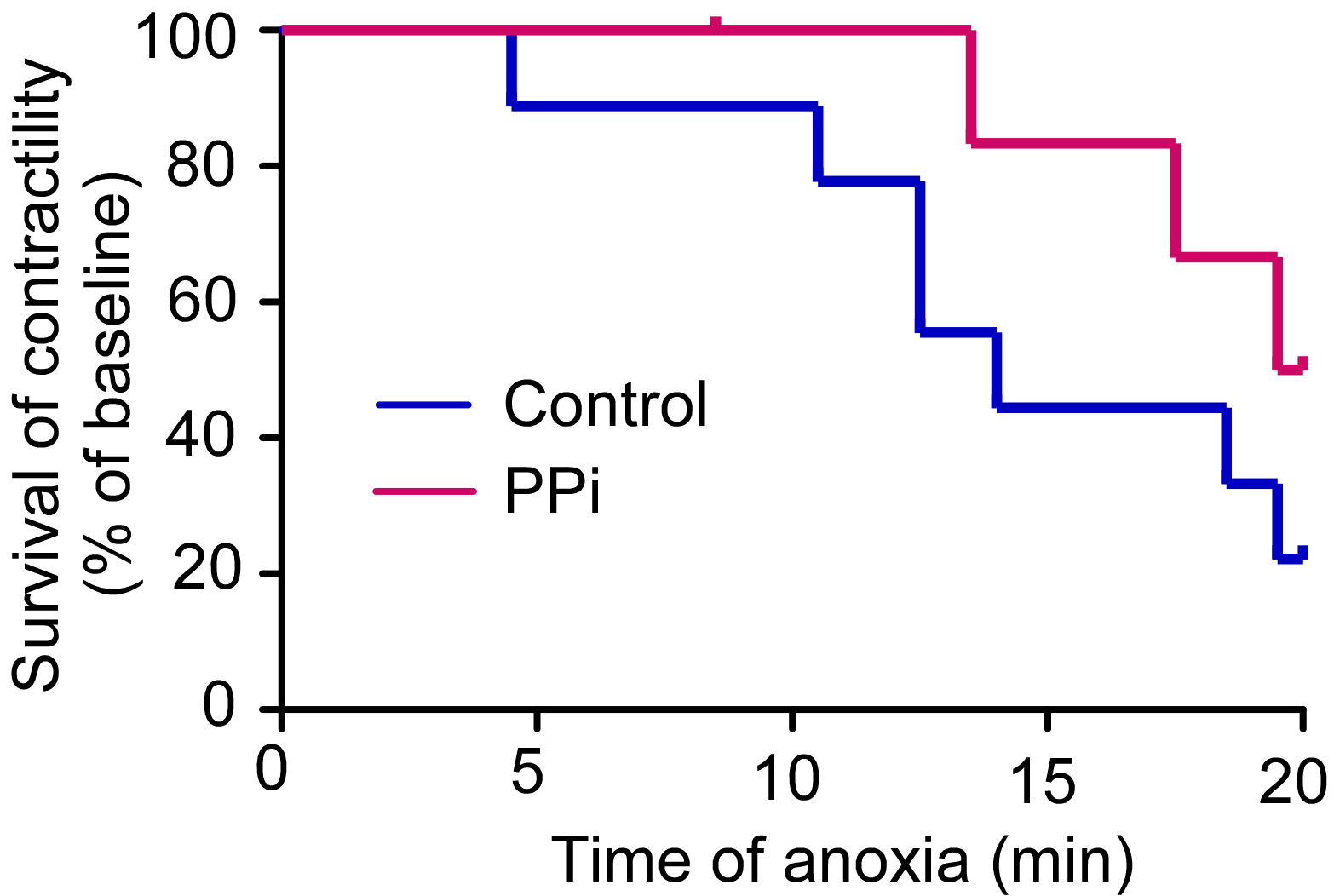

Supplement: Figure S8 — Effect of PPi on maintenance of contractility during simulated-demand ischemia in adult myocytes. Cellular contractility during anoxia+tachypacing was maintained longer in the PPi-treated cells (red line) than in control cells (blue line) although the difference did not reach statistical significance by log-rank test. Yet it should be noted that at 20 min of anoxia+tachypacing, only 22% of control myocytes maintained contractility, while in PPi group 50% of myocytes maintained contractility at this time point. (TIF) [file pone.0057821.s008.tif]
