# Supplementary material for: Metabolic Determinants of Electrical Failure in Ex-Vivo Canine Model of Cardiac Arrest: Evidence for the Protective Role of Inorganic Pyrophosphate
Source: PLoS One. 2013 Mar 8;8(3):e57821. doi: 10.1371/journal.pone.0057821 (PMC3592894; doi:10.1371/journal.pone.0057821)
Supplement: Table S1 — Tissue sampling time. (DOC) [file pone.0057821.s009.doc]

**Table S1:** **Tissue sampling time (min ± SEM)**
