# Supplementary material for: Metabolic Determinants of Electrical Failure in Ex-Vivo Canine Model of Cardiac Arrest: Evidence for the Protective Role of Inorganic Pyrophosphate
Source: PLoS One. 2013 Mar 8;8(3):e57821. doi: 10.1371/journal.pone.0057821 (PMC3592894; doi:10.1371/journal.pone.0057821)
Supplement: Table S2 — Animal profile and preparation time for hearts in control group. (DOC) [file pone.0057821.s010.doc]

**Table S2: Animal profile and preparation time for hearts in *control* group (± SEM)**
