# Supplementary material for: Metabolic Determinants of Electrical Failure in Ex-Vivo Canine Model of Cardiac Arrest: Evidence for the Protective Role of Inorganic Pyrophosphate
Source: PLoS One. 2013 Mar 8;8(3):e57821. doi: 10.1371/journal.pone.0057821 (PMC3592894; doi:10.1371/journal.pone.0057821)
Supplement: Text S1 — Detailed methods. (DOC) [file pone.0057821.s011.doc]

**Text S1: Detailed Methods**

The experimental protocol conformed to the *Guide for the Care and Use of Laboratory Animals* (The National Academy Press, 8^th^ edition, 2010) and was approved by University of Utah IACUC. For convenience, the full description of Methods is presented here, which includes everything given in the main text.

***Blood-perfused ex-vivo heart preparation***

Adult mongrel dogs of either sex (25.0 ± 1.3 kg) were premedicated with acepromazine (0.1 mL/10 kg, i.v.) and anesthetized with sodium pentobarbital (32.5 mg/kg). Under deep anesthesia maintained with pentobarbital the chest was opened via medial sternotomy and one jugular vein and one carotid artery were cannulated. One litter of blood was collected from a carotid artery concurrently with infusion of a Tyrode's solution (1 litter, composition in mmol/g: KCl 4.0, NaCl 130, CaCl_2_ 1.8, MgCl_2_ 1.0, NaHCO_3_ 24, NaH_2_PO_4_ 1.2, glucose 5.6; insulin 8 IU)[1] into a jugular vein. After that the heart was quickly removed from the chest and connected to a Langendorff perfusion apparatus. The heart was perfused with blood-Tyrode’s mixture which was filtered, heated and oxygenated (95%0_2_/5%CO_2_) using standard pediatric heart perfusion units from various vendors at a flow rate approximately 1ml per gram heart weight per minute. All the outflow of the perfusate was collected for recirculation via drainage tubes inserted into the RV and LV via cuts in the appendages of the respective atria. The heart was immersed in a temperature-controlled bath filled with warmed Tyrode’s solution, which was continuously pumped, without recirculation, at a rate 120-150 ml/min. The oxygen content in the superfusate was minimized by continuous gassing with 95% N_2_/5% CO_2_. The temperatures in the left ventricular cavity and superfusate were maintained at 37 ± 0.5 ^o^C during both normal coronary perfusion and no-flow ischemia. The gradient of temperature across the left ventricular wall did not exceed 1^o^C. Approximately 3 minutes before the induction of ischemia, the perfusion was switched to warm (37°C) oxygenated (95%0_2_/5%CO_2_) Tyrode’s solution with different concentrations of glucose and insulin depending on the experimental protocol (see below). Ventricular fibrillation was induced by a brief (~ 1 second) application of a 9V battery to the RV outflow tract. No-flow ischemia was initiated by stopping aortic perfusion which occurred approximately 10 seconds after VF induction, to ensure that VF was sustained. Global ischemia was maintained for 20 min. In all experiments the time of LDVF was counted from the moment when perfusion was stopped.

***Manipulations on glycolytic substrates and experimental groups***

In *control* group (n=9) no substrate manipulations were performed during ~90 minutes preceding LDVF and the pre-LDVF Tyrode’s solution contained normal concentration of glucose (5.5 mmol/L) and no insulin. In triple glucose group (*3x-glucose*, n=6) the pre-LDVF Tyrode’s solution contained three times the concentrations of glucose concentrations in controls (16.5 mmol/L) plus insulin (8 IU/L). In the glucose supplement group (*glu-suppl*, n=4) glucose level in the blood-Tyrode mixture was maintained at ~5.5 mmol/L for 90 minutes prior to LDVF by monitoring glucose level using a glucometer (Contour, Bayer HealthCare, Mishawaka, IN) every 10 minutes and supplying a glucose bolus (1g/ml in Tyrode’s solution) as necessary. The pre-LDVF Tyrode’s solution, however, contained normal glucose concentration (5.5 mmol/L) as in *control*. As a result, the level of glycogen at the onset of LDVF was increased (see Figure S5 below) while the pre-LDVF level of glucose was normal.

The limited size of *glu-suppl* group was due to circumstances beyond our control and related to an abrupt and unforeseen interruption in the availability of adult, random source dogs used in this study. While it was not possible to increase the number of *glu-suppl* hearts for the purposes of metabolic analysis, we thought it was appropriate to augment the electrophysiological aspect of this study using electrical data from 7 hearts used in a different, yet unpublished study. These hearts were subjected to the same experimental protocol but without tissue sampling. We will refer to this “external” group as *glu-suppl-ex*.

In *defib* group (n=6) monophasic defibrillation shocks were delivered between two electrodes immersed in the superfusion bath and positioned at a distance of approximately 1 cm from the left ventricular and right ventricular lateral free wall. Shocks were applied after one minute of VF in normally perfused hearts and after 15-20 minutes of LDVF. The shocks were applied at increasing strengths of 5, 10, 20, 30 and 50 J and were repeated up to 3 times at 50 J.

In addition to experimental groups determined by different experimental conditions (*control*, *3x-glucose*, *glu-suppl* and *glu-suppl-ex*) we defined *a-posteriori* early- and late-asystole groups*,* using *K-means* cluster analysis. This approach was first applied to the *control* group (n=9) yielding *control-early-asys* (n=4) and *control-late-asys* (n=5) groups. Because the notion of bimodal outcome appeared to be present regardless of substrate manipulations, the same approach was applied to the union of all groups where both electrical and metabolic data were available (*control* + *3x-glucose* + *glu-suppl,* total n=19*)*, yielding *all-early-asys* (n=10) and *all-late-asys* (n=9) *a-posteriori* groups. Lastly, in *defib* group the hearts which were in asystole at the time of shock application were designated as *defib-early-asys* group, and those which were in VF at the time of shock were designated as *defib-late-asys* group.

***Electrical recordings***

Volume-conductor ECG was recorded from two electrodes attached to the walls of the superfusion chamber with the ground electrode attached to the bottom of the chamber (in the first 4 experiments) and subsequently to the connective tissue surrounding the aorta. To record local electrical activity, plunge needle electrodes with 10 evenly spaced unipolar leads (inter-lead, distance, 1.6 mm) were manufactured in-house following a design developed by Rogers et al.[2] Three needles were placed in anterior left ventricle (LV) free wall (~20 mm apart), four needles were located in anterior right ventricle (RV) free wall, and three needles were inserted in the inter-ventricular septum along the left anterior descending (LAD) coronary artery*.* Ventricular fibrillation rate (VFR) was analyzed in LV mid-myocardial recordings, since they were the closest to the sites from which the tissue samples were taken, but were not disturbed significantly by tissue removal as is the case for epicardial and subepicardial LV leads. The other LV recordings as well as those from RV and the septum were however inspected in all experiments to verify the event of VF termination and the onset of global asystole or severe bradycardia. Asystole/severe bradycardia was defined as spontaneous VF termination observed in all recordings followed by at least 15 sec of complete electrical silence and a rhythm with rate <20 bpm for at least one minute thereafter. This definition encompasses a wide range of extremely slow and irregular rhythms observed after VF termination; in most cases however the rhythm was close to “pure” asystole with periods of complete silence spanning several minutes (see Figure S1). For that reason and for brevity we will refer to asystole/severe bradycardia as simply asystole.

***Electrode data analysis***

Unipolar electrograms were analyzed using custom software developed in a Matlab framework. Activations were chosen as the maximum negative derivatives that exceeded a minimum of -0.75 V/sec within a 20-ms search window, which is similar to approaches described previously [3]. VFR was calculated as an average number of activations per second over 10-second intervals taken at 10 seconds after VF induction and at minutes 1 to 20 of LDVF. For simplicity, the first time point will be referred to as 0 min LDVF. Since the epicardial surface was disrupted by tissue sampling, VFR from the LV mid-myocardial layer was presented in this study (Figure 1C).

***Tissue sampling***

Tissue samples were obtained from the upper 2-3 mm of anterior LV subepicardium in locations within 10 mm of at least one LV plunge needle electrode. Samples were taken at 0.3, 2, 5, 10, and 20 min after the onset of LDVF in all *control, 3x-glu* and *glu-suppl* hearts (see Table S1 for the average actual time of tissue sampling at each time point). In addition, baseline samples (before switching to Tyrode’s solution and induction of LDVF) were obtained in 5/9 *control* hearts, all 6 *3x-glu* hearts, and all 4 *glu-suppl* hearts. The tissue was excised quickly by cutting with sharp scissors around the plunge needle located in the mid LV free wall. The tissue samples were immediately frozen by liquid nitrogen and kept at -80°C until used. The freeze-clamped tissue samples were freeze-dried for at least 72 hours using a FreeZone plus 6 L freeze dryer (Labconco Corp., Kansas City, MO) to obtain a wet/dry tissue ratio, which was applied to the values measured by biochemical assays and HPLC for normalization [4].

***Metabolic Analysis***

*High Performance Liquid Chromatography (HPLC)*

The HPLC system was manufactured by Shimadzu and consisted of the following units, a SIL-10A XL autosampler, two LC-10 AD vp pumps, SPD-10A vp diode array detector and a CBM-10 AD vp controller, and a YMC-Pack ODS-A column (3.0 x 150 mm, Waters, Milford, MA). The system was operated and data collected using EZ start ver 7.4 software running on a Windows XP system. Mobile phase was prepared as described in Volonté et al.[5] except 2% acetonitrile and pH adjusted to 6.25 with KOH. The flow rate was 0.5 ml/min. Adenine nucleotides were detected at 259 nm whereas creatine compounds were detected at 209 nm (modified from Shellevold et al.[6]). The area of a peak was calculated in μmol/g by fitting into a standard curve (see below).

Standard Solutions: Standard stock solutions were prepared as described in Volonté et al.[5] Series of dilution were freshly made before each assay by adding the mobile phase, and calibration curves for all adenine nucleotides and creatine compounds were determined.

Preparation of Tissue Extracts: Tissue extracts were prepared as described in Shellevold et al.[6] Briefly, approximately 10-15 mg of frozen wet tissue was homogenized in 120 μl of 0.4 mol/L perchloric acid using a ceramic bead tube kit (MO BIO Laboratories, Inc., Carlsbad, CA) and FastPrep 24 (Krackeler Scientific, Inc, Albany, NY), which provides ultra-rapid shaking. After precipitation with1 mol/L KOH the extracts were centrifuged for 3 min at 4°C (13,000 g). The supernatant was transferred to a centrifugal filter (3,000NMWL; Millipore Corp., Billerica, MA) and spinned at 4°C (14,000 g). Twenty microliters of the filtered extracts was directly injected to the HPLC system.

*Gas chromatography/mass-spectrometry (GC/MS)*

Sample preparation: The freeze-clamped tissue samples (~5 mg) were homogenized in the extraction solution (methanol/water (8:1) with amino-acid standards) using ceramic bead lysis described above (speed: 6.5, time: 40 sec). Those homogenates were incubated for one hour at -20°C. After centrifugation (13,000 x g at 4°C for 10 min), the supernatant was centrifuged to remove large molecules. The extraction samples were dried using speed-vac (MiVac Duo, Barnstead/Genevac Inc., Gardiner, NY) overnight and derivatized using equal parts bistrimethyl-silyltriflouroacetamide and solvent mixture cetonitrile:dichloromethane:cyclohexane (5:4:1) with 5% triethylamine at 60ºC for 1 hour. Samples were maintained at 4ºC throughout the extraction process.

GC/MS analysis: All GC/MS analysis was performed with a Waters GCT Premier mass spectrometer fitted with an Agilent 6890 gas chromatograph as described by Shakoury-Elizeh et al.[7] Briefly, a Gerstel MPS2 autosampler was employed in conjunction with a CIS4 cold injection inlet. Dried samples were suspended in 40 uL of a 40 mg/mL O-methoxylamine hydrochloride in pyridine and incubated for one hour at 30°C followed by the transfer of 25 uL to autosampler vials. Ten uL of MSTFA was added by the autosampler and incubated for 30 minutes at 37°C with shaking. One uL of sample was injected to the cool inlet with the following program, 95°C for 30 seconds followed by a 10°C/second ramp to 250°C and a hold time of 3 minutes. The gas chromatograph held at an initial temperature of 95°C for one minute followed by a 40°C/min ramp to 110°C and a hold time of 2 minutes. This was followed by a second 5°C/min ramp to 250°C then a third ramp to 350°C with a final hold time of 3 minutes. A 30 m Restek Rxi-5 MS column with a 5 m long guard column was employed for analysis.

Data analysis: Data were collected by MassLynx 4.1. Initial analysis of known metabolites was performed using QuanLynx with data transfer to Excel. Peak picking was performed using MarkerLynx with data mining performed using SIMCA-P ver. 12.0.1. The values were normalized by wet tissue weight.

*Biochemical assays*

Myocardial glycogen content was determined as described in by Passonneau and Lauderdale [8]. Lactate content was assayed using the Lactate Assay kit II (BioVision, Inc., Mountain View, CA), in which the reaction is NADH-independent, to avoid the contamination by the increased intracellular NADH resulting from ischemia. NADtotal (NAD^+^ + NADH) and NADH were measured using NAD/NADH Quantitation Kit from the same vendor.

***∆Ψ measurement in rat neonatal myocytes***

*Cell culture*

Cellular experiments for ∆Ψm measurement were performed using Sprague-Dawley neonatal rats (1 day old). The hearts were removed under sterile conditions and dissociated using the Neonatal Cardiomyocyte Isolation kit (Worthington Biochemical Corporation, Lakewood, NJ). The isolated myocytes were then plated on a coverslip. All cells were incubated in the normal DMEM + horse serum at 37°C for the first 24 hours. After that, 0.1 mmol/L of PPi was added to DMEM + horse serum in the treatment group, whereas the control cells remained in the same normal media, and all cells were incubated for another 24 hours. All cells were used for experiment on the second day after isolation.

*∆Ψ measurement during near-anoxia*

The isolated myocytes cultured on coverslip were transferred into a bath and were superfused with the normal Tyrode’s solution (in mM: 126 NaCl, 4.4 KCl, 1.0 MgCl_2_, 11.0 glucose, 1.08 CaCl_2_, 24 HEPES, pH7.4) at 4ml/min (36 ± 0.6 ºC). The solution for the PPi-treated cells had the same composition, except the addition of 0.1 mM of PPi. The anoxic solution was modified from Bright et al. [9] First, the hypoxic solution was produced by replacing glucose with equimolar concentration of 2-deoxyglucose, warming up to 37ºC and gassing with 100% nitrogen for at least an hour. Aoxic solution used for the PPi group contained PPi (0.1 mM). In order to achieve near-anoxic conditions, 0.5 mM of oxygen scavenger sodium dithionite (Sigma, St. Louis, MO) was added directly to the hypoxic solution prior to its use. Stainless steel tubing was used to prevent diffusion of O_2_ into the anoxic solution. To prevent O_2_ diffusion from the air, 100% nitrogen gas was blown to the bath, which was covered by a custom-made coverslip. Partial oxygen pressure (pO_2_) in the bath solution was measured using ABL5 gas analyzer (Radiometer Copenhagen, Denmark). In the normal solution pO_2_ was ~175 mmHg, whereas in anoxic solution pO_2_ was 0 mmHg (undetectable). The replacement of normal solution with anoxic solution occurred within one second.

Then, potentiomeric fluorescent probe TMRM (0.4 µM) was added to the bath, and the cells were incubated with the dye approximately for 5 min. The TMRM fluorescence was excited at 543 nm and measured above 560 nm using Zeiss LSM 5 confocal microscope (Carl Zeiss, Jena, Germany). In order to limit phototoxicity and dye bleaching the laser exposure and recordings were performed for 4.0 ± 1.3 seconds at 60-second intervals during 5 minutes of baseline conditions and 10 minutes of anoxia. The cells in which the TMRM fluorescence decreased by more than 15% during 5 min of recording prior to anoxia were excluded from analysis. In order to quantify ∆Ψchanges before and during anoxia, the whole cell except nuclei was selected, and then the total TMRM fluorescence within non-nuclear part of cell interior was collected throughout the anoxic episode using ImageJ software and was normalized to the baseline value recorded immediately before the onset of anoxia.

***∆Ψm measurement in rabbit adult ventricular myocytes***

*Myocyte isolation*

Adult ventricular myocytes were isolated from rabbit (1.8 – 2.5 kg) hearts, as previously described [10,11]. Animals were anesthetized with pentobarbital sodium (50 mg/kg ip), then the excised heart was attached to an aortic cannular and then perfused at 37°C with the following sequence of solutions: Ca^2+^-free solution for 5 min to wash out the blood, low- Ca^2+^ (0.1 mM) solution containing 0.075 mg/ml of collagenase P (Roche Diagnostics, Indianapolis, IN) and 0.05 mg/ml protease (type XIV, Sigma Chemical, St. Louis, MO) approximately for 13 min, and enzyme-free solution containing 0.1 mM of CaCl_2_ for 5 min. The left ventricle was minced and shaken for 10 min in low- Ca^2+^ (0.1 mM) solution and then filtered through a nylon mesh. After Ca^2+^ concentration was adjusted to 1 mM, myocytes were stored at room temperature in a normal N-2-hydroxyethylpiperazine-N′-2-ethanesulfonic acid (HEPES)-buffered solution (in mM) 126 NaCl, 4.4 KCl, 1.0 MgCl_2_, 1.1 CaCl_2_, 11 dextrose, and 24.0 HEPES (free acid), titrated to pH 7.40 with NaOH). For PPi-group, the myocytes were incubated for 5 -6.5 h with 0.1 mM of PPi in the normal HEPES-buffer solution at room temperature. All myocytes used in this study were rod-shaped, had well-defined striations, and did not spontaneously contract.

*Cell solution and perfusion*

Myocytes were placed in a 1-ml flow-through chamber mounted on the stage of Zeiss confocal microscope. The coverslip on the bottom of the chamber was coated with laminin to enhance cellular adhesion. Myocytes were perfused with bathing solutions that flowed continuously through the bath at ~4–5 ml/min, and solution depth was held at ~2–3 mm. The control bathing solution was the HEPES-buffered solution described above. The bathing solution for PPi-treated cells contained 0.1 mM of PPi, which was added to a normal HEPES-buffered solution. Due to Ca- and Mg-binding properties of PPi, the final concentrations of Ca^2+^ and Mg^2+^ in the perfusion solution containing PPi decreased by 7.5 % and 5.1 %, respectively. The differences were considered too small to require adjustment. The anoxic solution was prepared by replacing glucose with 2-deoxyglucose in a normal HEPES-buffer solution, gassing with 100% nitrogen and adding sodium dithionite as described above. The temperature of the solutions in the bath were 37.0 ± 1.0°C.

*∆Ψm measurement in adult ventricular myocytes during tachypacing and anoxia*

The measurement of ∆Ψm was performed using TMRM fluorescence as described above. Myocytes from both control and PPi groups were paced at the cycle length (CL) of 280 ms for 5 min in the normal HEPES-buffer solution (tachypacing). After that myocytes were exposed to the anoxic solution, as described above, for 20 min, while continuously paced at CL=280ms (tachypacing+anoxia). This pacing CL was the shortest to allow all-or-none 1:1 capture under the conditions of this experiment; thus it was the closest possible CL to emulate the conditions of ischemic VF/VT present during cardiac arrest. Three-second recordings were taken at 60-second intervals during 5 minutes of tachypacing and 20 minutes of tachypacing+anoxia. ∆Ψm was measured using TMRM as described above. To quantify ∆Ψ changes, electrical stimulus was temporarily stopped during each recording (for ~3 sec), and the total TMRM fluorescence was measured in the region encompassing the entire cell except nuclei using ImageJ software. The measurements were performed during 25 min of recording (5 min of tachypacing and 20 min of tachypacing + anoxia) and normalized to the baseline value recorded at immediately before the initiation of tachypacing (at the time point “-5 min” in Figure 8E). The presence of cellular contractility was determined visually in cellular images taken every minute during tachypacing and tachypacing+anoxia protocols with pacing stimuli turned on.

***Statistical Analysis***

*K-means* cluster analysis was used to determine the optimal separation between the early asystole and the late asystole post-hoc groups. Timing of asystole was compared between *control*, *3x-glu,* and combined (*glu-suppl + glu-suppl-ex*) groups using log-rank test applied to Kaplan-Meier survival curves. For all comparisons involving multiple time points we used 2-way repeated measures ANOVA with post-hoc Bonferroni test. Otherwise, two-tailed unpaired t-test was used. A value of p<0.05 was considered statistically significant. Data are mean ± SEM.

***References***

1. Downar E, Janse MJ, Durrer D (1977) The effect of acute coronary artery occlusion on subepicardial transmembrane potentials in the intact porcine heart. Circulation 56:217-224

2. Rogers JM, Melnick SB, Huang J (2002) Fiberglass needle nlectrodes for transmural cardiac mapping. IEEE Transact Biomed Eng 49:1639-1641

3. Venable PW, Taylor TG, Shibayama J, Warren M, Zaitsev AV (2010) Complex structure of electrophysiological gradients emerging during long-duration ventricular fibrillation in the canine heart. Am J Physiol Heart Circ Physiol 299:H1405-1418

4. Cross HR, Opie LH, Radda GK, Clarke K (1996) Is a high glycogen content beneficial or detrimental to the ischemic rat heart? A controversy resolved. Circ Res 78:482-491

5. Volonte MG, Yuln G, Quiroga P, Consolini AE (2004) Development of an HPLC method for determination of metabolic compounds in myocardial tissue. J Pharm Biomed Anal 35: 647-653

6. Sellevold OF, Jynge P, Aarstad K (1986) High performance liquid chromatography: A rapid isocratic method for determination of creatine compounds and adenine nucleotides in myocardial tissue. J Mol Cell Cardiol 18: 517-527

7. Shakoury-Elizeh M, Protchenko O, Berger A, Cox J, Gable K, et al. (2010) Metabolic response to iron deficiency in saccharomyces cerevisiae. J Biol Chem 285:14823-14833

8. Passonneau JV, Lauderdale VR (1974) A comparison of three methods of glycogen measurement in tissues. Anal Biochem 60:405-412

9. Bright CM, Ellis D (1992) Intracellular ph changes induced by hypoxia and anoxia in isolated sheep heart purkinje fibres. Exp Physiol 77: 165-175

10. Warren M, Spitzer KW, Steadman BW, Rees TD, Venable P, et al. (2010) High-precision recording of the action potential in isolated cardiomyocytes using the near-infrared fluorescent dye di-4-anbdqbs. Am J Physiol Heart Circ Physiol 299: H1271-1281

11. Zaniboni M, Pollard AE, Yang L, Spitzer KW (2000) Beat-to-beat repolarization variability in ventricular myocytes and its suppression by electrical coupling. Am J Physiol Heart Circ Physiol 278:H677-687
